# Hitting Times of Some Critical Events in RNA Origins of Life

**DOI:** 10.3390/life11121419

**Published:** 2021-12-17

**Authors:** Caleb Deen Bastian, Hershel Rabitz

**Affiliations:** 1 Program in Applied and Computational Mathematics, Princeton University, Princeton, NJ 08544, USA; hrabitz@princeton.edu; 2 Department of Chemistry, Princeton University, Princeton, NJ 08544, USA

**Keywords:** RNA world, stochastic simulation algorithm, random counting measure, measure-kernel-function, ordinary differential equation, high dimensional model representation, global sensitivity analysis, fitness and similarity sequence landscapes, hitting times, survival analysis

## Abstract

Can a replicase be found in the vast sequence space by random drift? We partially answer this question through a proof-of-concept study of the times of occurrence (hitting times) of some critical events in the origins of life for low-dimensional RNA sequences using a mathematical model and stochastic simulation studies from Python software. We parameterize fitness and similarity landscapes for polymerases and study a replicating population of sequences (randomly) participating in template-directed polymerization. Under the ansatz of localization where sequence proximity correlates with spatial proximity of sequences, we find that, for a replicating population of sequences, the hitting and establishment of a high-fidelity replicator depends critically on the polymerase fitness and sequence (spatial) similarity landscapes and on sequence dimension. Probability of hitting is dominated by landscape curvature, whereas hitting time is dominated by sequence dimension. Surface chemistries, compartmentalization, and decay increase hitting times. Compartmentalization by vesicles reveals a trade-off between vesicle formation rate and replicative mass, suggesting that compartmentalization is necessary to ensure sufficient concentration of precursors. Metabolism is thought to be necessary to replication by supplying precursors of nucleobase synthesis. We suggest that the dynamics of the search for a high-fidelity replicase evolved mostly during the final period and, upon hitting, would have been followed by genomic adaptation of genes and to compartmentalization and metabolism, effecting degree-of-freedom gains of replication channel control over domain and state to ensure the fidelity and safe operations of the primordial genetic communication system of life.

## 1. Introduction

The origins of life, abiogenesis, is a matter of high importance, for it gives insight into the distribution of life in the universe. We focus on the RNA world hypothesis, where life began with self-replicating RNA molecules that can evolve under Darwinian evolution, following necessary conditions of compartmentalization and metabolism, for geometry and synthesis of nucleobases from metabolic precursors, respectively. Self-replicating sets of RNA were proposed first by Tibor Ganti [1,2] and have been studied by many others [3,4,5,6]. This is an information-centric perspective on abiogenesis, representing the putative beginning of genomic Darwinian evolution. Information centrism interprets a living organism as an operating genetic communication system in some connected domain that encodes and decodes genomic state relative to a replication channel.

While genomics, epigenomics, and transcriptomics of modern-day organisms are based on DNA, RNA, and epigenetic marks such as DNA methylation, RNA origins in their purest form concern the dual-function of RNA as an informational polymer and ribozyme. This article is similar in spirit to works in the 1970s through the 1990s, including Manfred Eigen’s works on replicating sets of RNA [7,8]. Clues to the RNA world, among others, are found in the nucleotide moieties in acetyl coenzyme A and vitamin B12, the structure of the ribosome as a ribozyme [9] and moreover the centrality of RNA to the translation system, and the existence of viroids [10]. A putative canonical RNA origins sequence involves RNA dependent RNA polymerase (RdRp) ribozyme, whose first gene is perhaps the Hammerhead (HH) ribozyme, enabling rolling-circle amplification of the sequence [11]. This setup is unnecessary, as the first role of RNA may not have been template-assisted polymerization but instead based on mechanisms of RNA recombination and networking [12]. If we assume the RdRp sequence is size 200 nucleotides, then there are $4^{200}\approx 10^{120}$ sequences. Starting from some population of interacting RNA molecules, we are interested in the times of first occurrence of critical events. Evolution seems to have concluded this search for a high-fidelity replicator in a fairly short period of time, i.e., within 400 million years of the Earth having a stable hydrosphere [5]. RdRp’s are known to be very ancient enzymes, are necessary to all viruses with RNA genomes, and have been proposed to have originated from junctions of proto-tRNAs relative to the context of a primitive translation system [13]. Recent work has shown that replicative RNA and DNA polymerases have a common ancestor of a RdRp [14]. Directed evolution, selecting on polymerization, is a potential way of identifying such a ribozyme. Directed compartmentalized self-replicating systems (RNA or DNA polymerases) mimicking prebiotic evolution have been demonstrated whereby polymerases are selected on their ability to replicate their own encoding gene [15]. Directed evolution has identified a RdRp that can replicate its evolutionary ancestor, an RNA ligase ribozyme; however, at increased activity, it has reduced fidelity and cannot maintain the integrity of its information [16].

A subtlety to the RNA origins argument is that template-directed polymerization by its nature requires two copies of the RdRp sequence, one for the polymerase and another for the template. This makes the RNA origins search extend until two copies are discovered. Cross reactions with other species also may influence the search time for the high-fidelity replicators. The clay mineral montmorillonite, which is common on Earth, can catalyze RNA oligomerization [17]; however, the utility of montmorillonite in these activities is not thought to be sufficient for origins, having been extensively studied [18,19]. Interestingly, it has been proposed that clay not only promotes origins, but constitutes it, which then later gave rise to RNA-based replication [20]; such mineral life as a genetic communication system has a high mutation rate and is degenerate.

Theories of abiogenesis study metabolism [21], cellular compartmentalization [22,23,24], hydrothermal vent chemical gradient energy [25], or hot springs [26,27,28], and so on, to define geochemical settings suitable for origins. These settings are compatible with RNA origins. Compartmentalization leading to selection on random sequences has been explored by studying environment forcing in hot springs and their effects on sequence identification [26,27,28], where the hypothesis is that fluctuations in environment forcing through cycling of wet, dry, and moist phases of lipid-encapsulated sequences subject sequences to combinatorial selection and identify structural and catalytic functions from the initial system state of random sequences. These functions include metabolic activity, pore formation, and structural stabilization. We assume the prebiotic molecular inventories of RNA and its precursors are provided by meteorites [29] and/or by Miller–Urey processes [30], such as from formamide [31] or many phase synthesis [32,33]. For energy and environmental factors, we consider a variant of Darwin’s “warm little pond”, where the putative environment for RNA origins of life is an icy pond with geothermal activity, a hot spring, or perhaps a hydrothermal vent: ice and cold temperature facilitate complexing of single strands into double strands and polymerization [34], and heat (energy) facilitates dissociation of double strands into single strands. More information on abiotic sources of organic compounds, mechanisms of synthesis and function of macromolecules, energy sources, and environmental factors can be found in the literature [35].

RNA origins have attracted many modeling efforts and analyses [36,37]. The concept of a self-replicating set of RNA molecules was initially studied by Manfred Eigen [7], wherein he studied the error-threshold of the critical fidelity to the main information. Two-dimensional spatial modeling has been applied in which reactions occur locally with finite diffusion, suggesting a spatially localized stochastic transition [38], simulated using Gillespie’s stochastic simulation algorithm (SSA) [39]. Another model is of autocatalytic sets of collectively reproducing molecules, which has been developed in reflexively autocatalytic food-generated (RAF) theory [40]. Various physics-based analyses have been conducted, such as in light of Bayesian probability, thermodynamics, and critical phenomena [41]. Systems of quasi-species based on the principle of natural self-organization called hypercycles employ non-equilibrium auto-catalytic reactions [8]. Nonlinear kinetic models for polymerization have been used to study the emergence of self-sustaining sets of RNA molecules from monomeric nucleotides [42]. Theoretical analysis has been conducted into RNA origins. Attention has been drawn to an evolving population of dynamical systems and how dynamics affect the error threshold of early replicators and possibly towards compartmentalization conveying hypercycles [43]. String-replicator dynamics have been studied and properties suggested to be necessary to RNA origins, including the ability to operate a functional genetic communication system and ecological and evolutionary stability [44,45,46]. A variety of pre-RNA worlds have been suggested, with RNA being preceded or augmented by alternative informational polymers, such as other nucleic acids [47], beta amyloid [48], polycyclic aromatic hydrocarbons [49], lipids [24], peptides [50], and so on. It seems that pre-RNA worlds existed independent of the RNA world in the sense that they are not ancestral to the RNA world, and that these worlds may have had non-trivial interactions with the RNA world.

A key concept of stochastic systems is that of a hitting time: the time of the first occurrence of some event. We develop a simple mathematical model at the sequence level to represent the synthesis and function of RNA molecules in order to gain insight into the hitting times of various critical events of RNA origins of life. The idea is to study the surface of hitting times in terms of the structure of the system. The model lacks many features of realism, such as sequence size variability, finite sources of “food” (activated nucleotides in our context), limited diffusion rates, poor system mixing, and so on, in order to concentrate on the process as a search problem. The notion of fitness landscapes has been studied extensively in evolutionary biology [51]. Landscape topology has been considered in an Opti-Evo theory, which assumes sufficient environmental resources and argues that fitness landscapes do not contain “traps” and globally optimal sequences form a connected level-set [52].

We describe the model in Section 2, where we define a replicating reaction network, whose random realizations are constructed using SSA. We describe hitting times as key random variables of interest and characterize polymerization as a transition kernel. In Section 3, we conduct and discuss simulation studies based on SSA, where we analyze the structure of the polymerase measures and the input–output and survival behaviors of the hitting times given the parameters of the system. In Section 4, we end with conclusions.

## 2. Materials and Methods

We define M={Adenine,Uracil,Guanine,Cytosine}, abbreviated as M={A,U,G,C}, corresponding to the RNA bases. We study the space of sequences having length *n*, that is, $E=M^{n}$ with space of possibilities (a σ-algebra) $E=2^{E}$, so that the pair (E,E) is the measurable space of all RNA sequences of length *n* with $\left|E\right|=4^{n}$. We let ν be a probability measure (distribution) on (E,E), giving the probability space triple (E,E,ν). Appendix A gives an overview of (E,E,ν). A related space, though not utilized in this article, is the space of all RNA sequences up to length *n*, $E^{*}=\cup_{i=1}^{n}H^{i}$ with $E^{*}=2^{E^{*}}$ and size $|E^{*}|=\frac{4}{3}\left(|E|-1\right)$. We denote the collection of non-negative E-measurable functions by $E_{\geq 0}$.

We build a simplified mathematical model for the time-evolution of a population of interacting RNA molecules in solution. Let $X_{t}$ be the population at time $t\in R_{\geq 0}$ with initial population $X_{0}$. $X_{t}$ is a multiset, that is, it is a set containing elements possibly with repeats. We assume that the system is well mixed and has access to an infinite source of activated nucleotides.

The complement of x∈E is denoted $x^{c}\in E$, attained using the base-pairing *A* with *U* and *C* with *G*. Let

(1) h(x,y)∈{0,1,…,n}forx,y∈E

be the Hamming distance between x,y∈E as the number of positions in *x* and *y* where the nucleotides differ. We have h(x,x)=0 and $h(x,x^{c})=n$.

### 2.1. Core Model

We describe the reaction network of the system below. We simulate trajectories of the system using the stochastic simulation algorithm (SSA) [39], to simulate exact trajectories for the evolution of stochastic reaction networks here. SSA forms a Markovian process, where the arrival of reactions follows a Poisson (point) process, and assumes that the reaction volume is well mixed and homogeneous, with all parts of the system accessible for reactions. Reactions across various disjoint volume elements of the system are dependent. We do not consider the effects of finite diffusion, which effects a length scale above which disjoint volume elements are effectively independent [38].

#### 2.1.1. System

We model the population of sequences which can form double-stranded helices, dissociate, and replicate with mutation with replicator fitness and sequence specificity. We interpret each system element as a set, either containing one element—a single-stranded sequence—indicated as {x}—or two elements, single and complementary stranded sequences, indicated as $\left\{x\right\}\cup \left\{x^{c}\right\}=\left\{x,x^{c}\right\}$, where $x,x^{c}\in E$ (double bracket notation indicates a collection, or set, of sets, i.e., {{x},{y},{z},⋯}). We define system elements as sets

$$\begin{array}{cc} \bar{E} & \equiv \{\{x\}:x\in E\}\\ \bar{F} & \equiv \{\left\{x,x^{c}\right\}:x\in E\}\\ \bar{G} & \equiv \bar{E}\cup \bar{F} \end{array}$$

with respective σ-algebras, $\bar{E}=2^{\bar{E}}$, $\bar{F}=2^{\bar{F}}$ and $\bar{G}=2^{\bar{G}}$. The sizes are $|\bar{E}|=4^{n}$ and $|\bar{F}|=4^{n}/2$, and $|\bar{G}|=3\times 4^{n}/2$. The set $\bar{E}$ contains single-stranded sequences, whereas the set $\bar{F}$ contains double-stranded sequences; the set $\bar{G}$ is the union of $\bar{E}$ and $\bar{F}$, containing both single and double stranded sequences. These sets are necessary to track the various sequences (single and double stranded). The reaction network of the system is given by

(2) $$\begin{array}{cc} x+x^{c} & \overset{k_{ds}}{\to }x\cup x^{c} \end{array}$$

(3) $$\begin{array}{cc} x\cup x^{c} & \overset{k_{ss}}{\to }x+x^{c} \end{array}$$

(4) $$\begin{array}{cc} x+y & \overset{k_{rep}\left(x,y\right)}{\to }x+y+y_{*}^{c}. \end{array}$$

and expressed in terms of sets

$$\begin{array}{cc} \left\{x\right\},\left\{x^{c}\right\} & \overset{k_{ds}}{\to }\left\{x,x^{c}\right\}\\ \left\{x,x^{c}\right\} & \overset{k_{ss}}{\to }\left\{x\right\},\left\{x^{c}\right\}\\ \left\{x\},\{y\right\} & \overset{k_{rep}\left(x,y\right)}{\to }\left\{x\right\},\left\{y\right\},\left\{y_{*}^{c}\right\}. \end{array}$$

Reaction (2) is double-strand formation from complementary single-strands with reaction rate $k_{ds}$. Reaction (3) is the dissociation of double-strands into single-strands, caused by a heat source, with reaction rate $k_{ss}$. Reaction (4) is template-directed polymerization of a single-strand (the template) by another single-strand (the polymerase), producing a single-strand complementary to the template with some fidelity, with reaction rate $k_{rep}$ (which functionally depends on the polymerase and template sequences).

The polymerization reaction rate is defined by

(5) $$k_{rep}\left(x,y\right)=af\left(x\right)s\left(x,y\right)\in \left(0,a\right]\;\mathrm{for}\;x,y\in E$$

where a>0 is a positive constant, f:E↦(0,1] is the replicative *fitness* of *x* (as a polymerase) and s:E×E↦(0,1] is the *similarity* between *x* and *y*, a symmetric function. Finally, *x* replicates *y*, outputting a version $y_{*}^{c}$ with mutations, where each nucleotide position has fidelity probability p:E↦(0,1]. Note that the similarity function can be either trivial/constant, i.e., s(x,y)=1, or non-trivial. For example, if we assume sequence similarity to correlate to spatial proximity of sequences, as assumed below, then s(x,y) is non-trivial.

#### 2.1.2. High-Fidelity Set

To define a high-fidelity set, pick an arbitrary subset of sequences R⊂E as high-fidelity replicators of size r=|R|. We define R two ways.

We define R using a product of non-empty random nucleotide subsets $\left\{A_{i}\subseteq M:i=1,\ldots ,n\right\}$ for each nucleotide position

$$R=A_{1}\times \cdots \times A_{n}$$

so that $r=\prod_{i=1}^{n}\left|A_{i}\right|=1^{r_{1}}2^{r_{2}}3^{r_{3}}4^{r_{4}}$ where $r_{2}=|\{A_{i}:|A_{i}\left|=2\}\right|$, etc. and $r_{1}+r_{2}+r_{3}+r_{4}=n$. For simplicity, we assume $|A_{i}|\in \left\{1,4\right\}$ with fraction 4 being q∈(0,1). Thus, R is a subset of *E* defined as a product space.

For another construction of *R*, we define a finite union of *m* random sequences $R=\{x_{1},\ldots ,x_{m}\}$.

#### 2.1.3. Distance

We define the Hamming distance H between sequence *x* and high-fidelity sequence set R as

(6) H(x,R)=min{h(x,y):y∈R}∈{0,1,…,n}forx∈E.

#### 2.1.4. Fitness

We define “tent-pole” fitness $f_{k}$ of sequence x∈E and high-fidelity sequence set R for curvature parameter $k\in R_{\geq 0}$ as

(7) $$f_{k}\left(x,R\right)=\exp\left[-k\;H\left(x,R\right)\right]\in \left(0,1\right]\;\mathrm{for}\;x\in E.$$

The maximums are the sequences of the high-fidelity sequence set R, which are the “points” or “poles” of the surface, with exponential decay into the remainder of the space in string distance. The strength of the decay is governed by parameter *k*, called the curvature parameter, which can be specified through the value of fitness at H(x,R)=n (sequence dimension),

(8) $$k=-\frac{\log(f_{k}\left(x,R\right))}{n}$$

Appendix B describes other fitness functions.

#### 2.1.5. Similarity

We define two cases for the similarity function appearing in the reaction rate of template-directed polymerization. The first case is the trivial (constant) case where the

$$s_{b}\left(x,y\right)=b\in \left(0,1\right]\;\mathrm{for}\;\left(x,y\right)\in E\times E.$$

This assumes that there is no mechanism by which sequence specificity is selected for, such as in the case that polymerases should evolve to generically well replicate sequences, including their own. This means that they will spend much of their time replicating other sequences.

For the second case of a non-trivial similarity function, we note that RNA origins of life are thought to be a spatially localized stochastic transition, where high-fidelity replicators are found concentrated in foci, following from the increased replicative mass of the replicators. Hence, we implicitly encode spatial information through a non-trivial similarity function, based on a distance function, which increases the replicative system mass for similar (and here nearby) high-fidelity replicators, that is, if they’re similar, then they’re likely proximal. In the following definition, we use the notation ∧ for the minimum of two numbers, x∧y=min{x,y}. Distance *S* is defined between two sequences x,y∈E as

(9) $$S\left(x,y\right)=h\left(x,y\right)\wedge h\left(x,y^{c}\right)\wedge h\left(x^{c},y\right)\wedge h\left(x^{c},y^{c}\right)\in \left\{0,1,\ldots ,n\right\}\;\mathrm{for}\;x,y\in E.$$

Similarity $s_{k}$ of sequences x,y∈E for curvature parameter $k\in R_{\geq 0}$ is defined in terms of exponential decay as

(10) $$s_{k}\left(x,y\right)=\exp\left[-k\;S\left(x,y\right)\right)]\in \left(0,1\right]\;\mathrm{for}\;x,y\in E.$$

Presently, replicators can replicate other sequences well but not their own [34]. There may exist RdRps that are excellent polymerases and, in conjunction with RNA hammerhead ribozyme, engage in rolling circle amplification of the polymerase-hammerhead sequence (genome) so that the amplification process is self-cleaving. This results in a large increase in replicative mass due to the super-exponential growth in the population of the high-fidelity replicators within a small volume. In the context of SSA, the similarity function here is an ansatz for spatial locality.

#### 2.1.6. Fidelity

Finally, polymerization fidelity probability for curvature $k\in R_{\geq 0}$ is defined as

(11) $$p_{k}\left(x,R\right)=f_{k}\left(x,R\right)\in \left(0,1\right]\;\mathrm{for}\;x\in E.$$

Note that $f_{k}\left(x,R\right)=p_{k}\left(x,R\right)=1$ for high-fidelity sequences x∈R.

Note that fitness, similarity, and fidelity are defined for single-stranded sequences (E,E).

#### 2.1.7. Counting Representation

The process $X={\left(X_{t}\right)}_{t\in R_{\geq 0}}$ is the time-evolution of the system. Recall that $X_{t}$ is a multiset. $X_{t}$ contains the individual single stranded molecules in the set $\bar{E}$, i.e., sequences $\left\{x\right\}\in \bar{E}$ and double stranded molecules in the set $\left\{x,x^{c}\right\}\in \bar{F}$, with overall set $\bar{G}=\bar{E}\cup \bar{F}$ having size $m=|\bar{G}\left|=3\right|\bar{E}|/2$. Note that, in the set representation, there is symmetry $\left\{x,x^{c}\right\}=\left\{x^{c},x\right\}$, so that the size of the double-stranded set is equal to $|\bar{F}\left|=\right|\bar{E}|/2$. The system evolution $X_{t}$ induces a random counting measure $N_{t}$ on the overall space of single and double stranded sequences $\left(\bar{G},\bar{G}\right)$ as

(12) $$N_{t}\left(A\right)=\sum_{x\in X_{t}}I_{A}\left(x\right)\;\mathrm{for}\;A\in \bar{G}$$

The total count, that is, the total number of molecules, is $K_{t}\equiv \left|X_{t}\right|=N_{t}\left(\bar{G}\right)$. We assume that the counter $N_{t}$ is maintained for all times $t\in R_{\geq 0}$.

#### 2.1.8. Reaction Rates

The total reaction rate is given by the sum of the individual reaction rates

$$\overset{˘}{k}\left(t\right)={\overset{˘}{k}}_{ds}\left(t\right)+{\overset{˘}{k}}_{ss}\left(t\right)+{\overset{˘}{k}}_{rep}\left(t\right)\;\mathrm{for}\;t\in R_{\geq 0}$$

where the reaction rate of double-strand formation from complementary single-strands is given by

$${\overset{˘}{k}}_{ds}\left(t\right)=\frac{1}{2}\sum_{\left\{x\right\}\in \bar{E}}k_{ds}N_{t}\left(\left\{x\right\}\right)N_{t}\left(\left\{x^{c}\right\}\right),$$

the reaction rate of dissociation of double-strands into single-strands is given by

$${\overset{˘}{k}}_{ss}\left(t\right)=\sum_{\left\{x,x^{c}\right\}\in \bar{F}}k_{ss}N_{t}\left(\left\{x,x^{c}\right\}\right),$$

and the reaction rate for template-directed polymerization of a single-strand by another single-strand (the polymerase) is given by

$${\overset{˘}{k}}_{rep}\left(t\right)=\sum_{\left(\left\{x\right\},\left\{y\right\}\right)\in {\bar{E}}^{2}}k_{rep}\left(x,y\right)N_{t}\left(\left\{x\right\}\right)\left(N_{t}\left(\left\{y\right\}\right)-I_{}\left(x=y\right)\right)$$

The first reaction rate is a sum over the single-strands of $\bar{E}$ with size $4^{n}$. The second is a sum over double-strands $\bar{F}$ with size $4^{n}/2$. The third reaction is a sum over the product space of single stranded sequences, ${\bar{E}}^{2}$, with $4^{2n}=16^{n}$ number of elements. Therefore, $\overset{˘}{k}\left(t\right)$ requires $4^{n}\left(\frac{3}{2}+4^{n}\right)$ elements to be evaluated for every reaction. Clearly, direct representation on the full space is very expensive and impractical for even modest *n*. One obvious way to improve efficiency is not summing over the zero elements. We define sets

(13) $$X_{1}=\{x\in \mathrm{set}\left(X_{t}\right):|x|=1\}$$

and

(14) $$X_{2}=\{x\in \mathrm{set}\left(X_{t}\right):|x|=2\}$$

as the unique single and double stranded sequences of the system. Then, direct calculations of the reaction rates are

$${\overset{˘}{k}}_{ds}\left(t\right)=\frac{1}{2}\sum_{\left\{x\right\}\in X_{1}}k_{ds}N_{t}\left(\left\{x\right\}\right)N_{t}\left(\left\{x^{c}\right\}\right)$$

and

$${\overset{˘}{k}}_{ss}\left(t\right)=\sum_{\left\{x,x^{c}\right\}\in X_{2}}k_{ss}N_{t}\left(\left\{x,x^{c}\right\}\right)$$

and

$${\overset{˘}{k}}_{rep}\left(t\right)=\sum_{\left(\left\{x\right\},\left\{y\right\}\right)\in X_{1}^{2}}k_{rep}\left(x,y\right)N_{t}\left(\left\{x\right\}\right)\left(N_{t}\left(\left\{y\right\}\right)-I_{}\left(x=y\right)\right).$$

For this approach, the replication rate has quadratic dependence on $|X_{1}|$. Using the reaction rates, the system may be exactly simulated using SSA. The reaction at time *t* with rate $\overset{˘}{k}\left(t\right)$ occurs over time interval $▵t∼\mathrm{Exponential}(1/\overset{˘}{k}\left(t\right))$. As the reaction rate $\overset{˘}{k}\left(t\right)$ increases with increasing number of molecules $K_{t}=N_{t}\left(\bar{G}\right)$, the reaction rate increases and reaction duration ▵t decreases over time. The natural consequence of increasing process intensity is that the system speeds up.

The quadratic dependence may still be too expensive for large simulations. Appendix E describes Monte Carlo approximation of the reaction rates.

### 2.2. Hitting Times

We define some hitting times. The initial population consists of *I* single-stranded sequences $X_{0}$, i.e., $|X_{0}|=I$. We define the hitting time τ for the time of the first replication event

(15) $$\tau_{\mathrm{rep}}=\inf\left\{t\in R_{\geq 0}:K_{t}>I\right\}.$$

We define the hitting time τ for the appearance of sequences in the high-fidelity sequence set R,

(16) $$\tau_{R}=\inf\{t\in R_{\geq 0}:I_{\left\{1,2,\cdots \right\}}(|R\cap X_{t}|)=1\}.$$

Put $X_{R}=R\cup \left\{\left\{x,x^{c}\right\}:x\in R\right\}$ and define the volume fraction of high-fidelity sequences R at time *t* as

(17) $$V\left(t\right)=\frac{N_{t}\left(X_{R}\right)}{K_{t}}.$$

We define the hitting time τ where high-fidelity sequences of R emerge and reach a minimum volume fraction,

(18) $$\tau_{\min}=\inf\left\{t\in R_{\geq 0}:t\geq \tau_{R},V\left(t\right)\leq V\left(s\right)\;\mathrm{for}\;\tau_{R}\leq s\leq t\right\}.$$

This hitting time reflects that period wherein a high-fidelity replicator has been identified yet there exists no complementary high-fidelity sequence for amplification, hence the system diversity continues to increase, decreasing the concentrations of all extant sequences as more sequences are discovered. The minimum hitting time captures the duration of time the high-fidelity replicator exists by itself. We define the hitting time τ for the time high-fidelity sequences in R constitutes some volume fraction v∈(0,1] of the population,

(19) $$\tau_{v}=\inf\left\{t\in R_{\geq 0}:V\left(t\right)\geq v\right\}.$$

In practice for simulations, $\tau_{v}$ is censored based on some total number of reactions, that is, if the volume fraction is not achieved by *n* reactions, $\tau_{v}=\infty$ because there is no arrival time.

For SSA, we specify a maximum number of reactions *N* to simulate. We have parameters θ∈Θ for τ, such as landscape curvature *k*, sequence dimension *n*, etc. Therefore, τ(θ) is right-censored with value *∞* at simulation time *a*, as some simulations will stop at time *a* with no arrival time. These are censoring events. For fixed θ, the τ(θ) is a random variable, due to the stochastic nature of SSA. Hence, for each parameter vector θ, we attain a set of *M* realizations of hitting time τ as

(20) $$T\left(\theta \right)=\{\tau_{i}\left(\theta \right):i=1,\ldots ,M\}.$$

For convenience, we assume that the realizations T(θ) are ordered by non-censored followed by censored.

#### 2.2.1. Functional Structure

For each parameter vector θ, we record two values: the number of hitting events in the hitting time set T

(21) g(θ)=|{x∈T(θ):x<∞}|∈{0,1,…,M}

and the average of the hitting time τ

(22) $$f\left(\theta \right)=\left\{\begin{array}{cc} \frac{1}{g(\theta )}\sum_{i=1}^{g(\theta )}\tau_{i}\left(\theta \right) & \mathrm{if}\;g(\theta )>0\\ 0 & \mathrm{if}\;g(\theta )=0 \end{array}\right.\in R_{\geq 0}$$

To describe the functional structure of the average hitting time f(θ), we require a classifier which determines whether or not there are zero hittings g(θ)=0 and a regressor for the value of f(θ) for hittings g(θ)>0. We assume that the parameters $\theta =(\theta_{1},\cdots ,\theta_{n})$ are randomly sampled according to distribution $\nu =\prod_{i}\nu_{i}$ and the hitting times recorded. High dimensional model representation (HDMR) may be attained for the classifier (as a probabilistic discriminative model) and the regressor of f(θ). For the regressor, we have HDMR expansion

$$f\left(\theta_{1},\cdots ,\theta_{n}\right)=f_{0}+\sum_{i}f_{i}\left(\theta_{i}\right)+\sum_{i<j}f_{ij}\left(\theta_{i},\theta_{j}\right)+\cdots +f_{1\cdots n}\left(\theta_{1},\cdots ,\theta_{1}\right)$$

The HDMR component functions $\left\{f_{u}\right\}$ convey a global sensitivity analysis, where, defining variance term

$$\sigma_{u}^{2}=Varf_{u}=\int_{\Theta }f_{u}^{2}\left(\theta_{u}\right)\nu \left(d\theta \right),$$

we have a decomposition of variance

$$\sigma_{f}^{2}=Varf=\sum_{u\subseteq \{1,\cdots ,n\}:|u|>0}\sigma_{u}^{2}$$

The normalized terms $S_{u}=\sigma_{u}^{2}/\sigma_{f}^{2}$ are called sensitivity indices. Appendix F gives a brief description of global sensitivity analysis via HDMR.

#### 2.2.2. Statistical Structure

A second analysis can be conducted on the hitting times T(θ) for the parameter vector θ using reliability theory. Put random hitting set T(Θ)≡{T(θ):θ∈Θ} where $\Theta =\{\theta_{i}\}$ is an independency of parameter values. For each parameter vector θ∈Θ, we partition the hitting times T(θ) into *C* censored values with censor times $C\left(\theta \right)=\{a_{i}\left(\theta \right)\}$ and M−C non-censored (hitting) values N(θ)={x∈T(θ):x<∞}. The likelihood is given by

$$L\left(T\left(\Theta \right)|\vartheta \right)=\prod_{\theta \in \Theta }\prod_{x\in N(\theta )}f\left(x|\vartheta \right)\prod_{x\in C(\theta )}R\left(x|\vartheta \right),$$

where *f* is the hitting time probability density function (‘failure density’) and *R* is censoring time distribution (‘reliability distribution’), and ϑ are the parameters of the density and distribution functions. Note that *f* and *R* each specify each other, so ϑ are the common parameters. Reliability definitions are given in Appendix G.

We show reliability quantities in Table 1 for the two-parameter $\left(\alpha ,\beta \right)\in {\left(0,\infty \right)}^{2}$ Weibull (α,β) distribution and Cox proportional hazard’s model where γ is a vector of coefficients for the θ. We use the Python software *lifelines* for estimation of ϑ for the Weibull–Cox model from data [53]. The mean failure time Eτ(θ) is given by

$$E\tau \left(\theta \right)=\int_{0}^{\infty }t\;f\left(t,\theta |\alpha ,\beta ,\gamma \right)=\beta e^{-\gamma \cdot \theta /\alpha }\Gamma \left(1+\frac{1}{\alpha }\right)$$

We have second moment

$$\int_{0}^{\infty }t^{2}\;f\left(t,\theta |\alpha ,\beta ,\gamma \right)=\beta^{2}e^{-2\gamma \cdot \theta /\alpha }\Gamma \left(1+\frac{2}{\alpha }\right)$$

giving variance

$$Var\tau \left(\theta \right)=\beta^{2}e^{-2\gamma \cdot \theta /\alpha }\left(\Gamma \left(1+\frac{2}{\alpha }\right)-\Gamma^{2}\left(1+\frac{1}{\alpha }\right)\right)$$

Thus, if γ<0, then Eτ(θ) and Varτ(θ) exponentially increase in θ.

### 2.3. Surface Chemistries

The system given by (2) of polymerization with mutation requires two separate hitting events, one sequence in the high-fidelity set x∈R and either another sequence in the high-fidelity set x∈R or its complement $x^{c}\in E$, in order for high-fidelity replicators to maximally engage in templated-directed polymerization and achieve some fraction of the population. This setup of RNA polymerase action, requiring two such events for the polymerase and template, makes the hitting times long. Basically, the same information must be discovered twice before it can be used, which is unsatisfactory. We idealize polymerase activity conveyed by a non-RNA species, here clay, with the parameter $k_{clay-p}$, as clay itself is not thought to be capable of polymerization but is capable of oligomerization of RNA. The reactions are given by

(23) $$\begin{array}{cc} ⌀ & \overset{k_{clay-o}}{\to }x \end{array}$$

(24) $$\begin{array}{cc} x & \overset{k_{clay-p}}{\to }x+x_{*}^{c} \end{array}$$

where non-RNA polymerization has mutation with fidelity probability p∈(0,1] and $x_{*}^{c}$ is the complement of *x* with mutation. The reaction rates are given by

$${\overset{˘}{k}}_{clay-o}\left(t\right)=k_{clay-o}$$

and

$${\overset{˘}{k}}_{clay-p}\left(t\right)=k_{clay-p}N_{t}\left(X_{1}\right)$$

Therefore, upon the first hitting of the high-fidelity replicators R with sequence x∈R through (4) or (23), *x* gives two high-similarity single-stranded sequences *x* and $x_{*}^{c}$ through (24), which then may participate in template-directed RNA polymerization (4).

### 2.4. Reactions as Measure-Kernel-Functions

All the reactions x↦y which involve substrate *x* may be represented using transition kernels, which form linear operators. At each iteration of SSA, a reaction type is chosen, followed by a transition to a particular domain (X,X) with distribution $\nu_{t}$, followed by mapping into a codomain (Y,Y) using transition probability kernel *Q* with distribution $\mu_{t}=\nu_{t}Q$. The notions of $\nu_{t}$ and *Q* involve measure-kernel-functions. The probability of transition of *y* into B∈Y given *x* is given by *Q*

$$Q\left(x,B\right)=\int_{B}Q\left(x,dy\right)=P\left(y\in B:x\right)$$

Appendix C recalls some facts about *Q*.

We define kernels *Q* for RNA and non-RNA polymerization to provide insight into the reactions. Consider $X_{t}$ for some $t\in R_{\geq 0}$. Recall that $N_{t}$ is the random counting measure of $X_{t}$ on single and double-stranded sequences $\left(E\cup F,2^{E\cup F}\right)$. For RNA and non-RNA polymerization, we take $\nu_{t}$ as a (random) probability measure for *x* in domain (X,X) and describe a transition probability kernel *Q* from *x* into *y* in codomain (Y,Y).

#### 2.4.1. RNA Polymerization

For RNA polymerization, we have that

$$\left\{x\right\},\left\{y\right\}\mapsto \left\{x\right\},\left\{y\right\},\left\{y_{*}^{c}\right\}$$

which results in the creation of the single-stranded sequence $\left\{y_{*}^{c}\right\}$. The first dimension is the polymerase and the second is the template. We give a definition of the probability measure on the product space of sequences. Recall that ${\left(E⊗E\right)}_{\geq 0}$ denotes the collection of non-negative E⊗E-measurable functions.

**Definition** **1** (Measure on domain $\nu_{t}$). *Let $\nu_{t}$ be a random probability measure on (E×E,E⊗E) formed by random counting measure $N_{t}$ (12)*

$$\nu_{t}\left\{x,y\right\}=\frac{k_{rep}\left(x,y\right)N_{t}\left(\left\{x\right\}\right)\left(N_{t}\left(\left\{y\right\}\right)-I_{}\left(x=y\right)\right)}{{\overset{˘}{k}}_{rep}\left(t\right)}\;\mathrm{for}\;\left(x,y\right)\in E\times E$$

*with*

(25) $$\nu_{t}\left(f\right)=\sum_{(x,y)\in E\times E}\nu_{t}\left\{x,y\right\}f\circ \left(x,y\right)\;\mathrm{for}\;f\in {\left(E⊗E\right)}_{\geq 0}$$

*We write $\nu_{t}\left(A\right)=\nu_{t}I_{A}$ *for* A∈E⊗E.*

Because the first two coordinates are preserved under the mapping, we focus on the new dimension as a transition from (E×E,E⊗E) into (E,E) using transition probability kernel *Q*. In this case, *Q* is defined by a $16^{n}\times 4^{n}$ matrix whose rows vectors (dimension $4^{n}$) are probability vectors. The structure of *Q* follows from the polymerase replication with mutation, whereby each nucleotide position has fidelity probability p:E↦(0,1], which depends on the first dimension of E×E. We put $p_{x}=p\left(x\right)$ for sequence x∈E. Now, we state a simple fact on the binomial structure of the number of mutations made by a polymerase.

**Theorem** **1** (Mutation distribution). *The number of mutations by polymerase x∈E on template y∈E is distributed*

$$h\left(y^{c},y_{*}^{c}\right)∼\mathrm{Binomial}\left(n,1-p_{x}\right)\;\mathrm{for}\;p_{x}\in \left(0,1\right)$$

*with mean $n(1-p_{x})$ and variance $np_{x}\left(1-p_{x}\right)$ and*

$$h\left(y^{c},y_{*}^{c}\right)∼\mathrm{Dirac}\left(0\right)\;\mathrm{for}\;p_{x}=1.$$

Now, we partition *E* into level sets $\left(H_{i}\left(y\right)\right)$ by Hamming distance to the template complement $y^{c}$,

(26) $$H_{i}\left(y\right)=\left\{x\in E:h\left(y^{c},x\right)=i\right\}\;\mathrm{for}\;i\in \left\{0,\ldots ,n\right\}.$$

We define the transition kernel *Q* for RNA polymerization, where *Q* completely encodes RNA polymerization using Theorem 1.

**Corollary** **1** (Transition probability kernel *Q*). *We have that the transition probability kernel Q for RNA polymerization is defined by*

Q((x,y),Hi(y))=ni(1−px)ipxn-ifor(x,y)∈E×E,i∈{0,…,n},px∈(0,1)

*and*

Q((x,y),{z})=1|Hi(y)|ni(1−px)ipxn−ifor(x,y)∈E×E,i={0,…,n}z∈Hi(y),px∈(0,1)

*and*

$$Q\left(\left(x,y\right),\left\{y^{c}\right\}\right)=1\;\mathrm{for}\;\left(x,y\right)\in E\times E,\;p_{x}=1.$$

RNA polymerization is defined by *Q* using the binomial structure of polymerase mutation. A more sophisticated model could be defined as a sum of Bernoulli random variables with varying success probabilities in the Poisson binomial distribution. This could be used to take into account polymerase mutation that varies with nucleotide position. Another idea is taking into account schemata such as repeats which destabilize the polymerase [54].

**Proposition** **1** (Measure on codomain $\mu_{t}$). *$\mu_{t}=\nu_{t}Q$ is a probability measure on (E,E) defined by*

(27) $$\mu_{t}\left(f\right)=\int_{E\times E}\nu_{t}\left(dx,dy\right)\int_{E}Q\left(\left(x,y\right),dz\right)f\left(z\right)\;\mathrm{for}\;f\in E_{\geq 0}$$

*It is multiplication of $\nu_{t}$ as a $16^{n}$ dimension row vector with $16^{n}\times 4^{n}$ dimension matrix Q, giving a $4^{n}$ dimension row vector $\nu_{t}Q$. We write $\mu_{t}\left(A\right)=\mu_{t}I_{A}$ for A∈E.*

Define the partition $\left(H_{i}\right)$ of *E* as

(28) $$H_{i}\equiv \left\{x\in E:\min\left\{H\left(x,R\right),H\left(x^{c},R\right)\right\}=i\right\}\;\mathrm{for}\;i\in \left\{0,\ldots ,n\right\}.$$

Then, $\mu_{t}\left(H_{i}\right)$ for i∈{0,…,n} is the distribution on sequences by distance to *R*, i.e.,

$$\mu_{t}\left(H_{i}\right)=\sum_{x\in E}\mu_{t}\left\{x\right\}I_{H_{i}}\left(x\right)\;\mathrm{for}\;i\in \left\{0,\ldots ,n\right\}$$

contains the instantaneous information of RNA polymerization.

A more general model for replication is where polymerase activity is tied to geometry, i.e., compartmentalization/spatial confinement, with state space (C,C) and to metabolic state (M,M). In this telling, the polymerase reaction rate could be tied to the degree of spatial confinement and the source of the activated nucleotides from metabolic precursors. Then, the polymerase state-space is (C×M×E×E,C⊗M⊗E⊗E) with law $\nu_{t}$ and the polymerase transition kernel $Q_{cme}$ is defined as the mapping from (C×M×E×E,C⊗M⊗E⊗E) into (E,E). Thus, the law on the input–output space-state (C×M×E×E×E,C⊗M⊗E⊗E⊗E) is given by $\mu_{t}=\nu_{t}\times Q_{cem}$, or in differential notation,

$$\mu_{t}\left(dc,dm,dx,dy,dz\right)=\nu_{t}\left(dc,dm,dx,dy\right)Q_{cem}\left(\left(c,m,x,y\right),dz\right)$$

#### 2.4.2. Non-RNA Polymerization

If there exists some kind of non-RNA polymerase activity, we have that the mapping

$$\left\{x\right\}\mapsto \left\{x\right\},\left\{x_{*}^{c}\right\}$$

which we regard as a mapping from (E,E) into (E,E). Let $\nu_{t}$ be a probability measure on (E,E) defined by

$$\nu_{t}\left\{x\right\}=\frac{N_{t}\left(\left\{x\right\}\right)}{N_{t}\left(E\right)}\;\mathrm{for}\;x\in E$$

Similar to RNA polymerization, for fidelity probability p∈(0,1], we have *Q* as the $4^{n}\times 4^{n}$ matrix defined by

Q(x,Hi(x))=ni(1−p)ipn−iforx∈E,i∈{0,…,n},p∈(0,1)

and

Q(x,{z})=1|Hi(x)|ni(1−p)ipn−iforx∈E,i={0,…,n},z∈Hi(x),p∈(0,1)

and

$$Q(x,\left\{x^{c}\right\})=1\;\mathrm{for}\;x\in E,\;p=1.$$

$\mu_{t}=\nu_{t}Q$ is a probability measure on (E,E) defined by

$$\mu_{t}\left(f\right)=\int_{E}\nu_{t}\left(dx\right)\int_{E}Q\left(x,dy\right)f\left(y\right)\;\mathrm{for}\;f\in E_{\geq 0}$$

Note that, for SSA, *Q* is fixed over the simulation, whereas the probability measure $\nu_{t}$ depends on time. That is, the reactions are chosen according to the reaction rates, and the reactions each use respective *Q*. The $\nu_{t}$ is formed using a random counting measure, so $\nu_{t}$ is random. This approach generalizes in the obvious way to all the reactions.

### 2.5. Decay

The RNA sequences have finite lifetimes in reality. This comes from a variety of sources, including radiation, pH, intrinsic molecular stability, etc. We assume double-stranded RNA is stable, whereas single-stranded RNA is not. Therefore, we create a reaction for decay of single-stranded RNA into constitutive nucleotides

(29) $$x\overset{k_{⌀}}{\to }⌀$$

with reaction rate

$${\overset{˘}{k}}_{⌀}\left(t\right)=k_{⌀}N_{t}\left(X_{1}\right)$$

### 2.6. Compartmentalization

It is thought that compartmentalization plays a role in RNA origins of life, giving foci of reproducing sequences [23,55]. This is somewhat anticipated by the similarity function s:E×E↦(0,1], where sequences are more likely to copy similar sequences than less similar ones, due to an underlying spatial localization. Explicit spatial effects may be modeled by assuming each x∈E is marked with a position on a bounded subset of the real line $\left(\left[-T,T\right],B_{\left[-T,T\right]}\right)\subset \left(R,B_{R}\right)$. We think of this as a one-dimensional projection of the three-dimensional system. Additional species can be introduced, such as lipids, with reactions forming a vesicle *M* (vesiculation), which encloses some A=[r,s]⊂[−T,T]. We assume the lipids interact with the single stranded sequences in *A* to form vesicles as

(30) $$x\overset{k_{mic}}{\to }M\left(A\right)$$

with reaction rate

$$k_{mic}\left(t\right)=k_{mic}N_{t}\left(X_{1}\right).$$

Hence, vesiculation is coupled to the population of sequences by design so that it evolves on roughly the same time-scale as sequence activities. Note that vesicles can enclose one another, i.e., M(A) and M(B) where A⊂B or B⊂A, but cannot cross, i.e., for all vesicles at locations A,…,B we have that A∩B∈{A,B,⌀}. For example, suppose one vesicle A=[0,1] encloses another two, $B=[\frac{1}{3},\frac{1}{2}]$ and $C=[\frac{2}{3},\frac{3}{4}]$. Then, $A\backslash \left(B\cup C\right)=\left[0,\frac{1}{3}\right)\cup \left(\frac{1}{2},\frac{2}{3}\right)\cup \left(\frac{3}{4},1\right]$. Although A\(B∪C) is disconnected in one dimension, the intervals are physically connected in three dimensions, where vesicles are spheres. We identify each vesicle to a union of disjoint intervals, disjoint across the vesicles.

We posit that compartmentalization precedes the hitting of a high-fidelity replicator through ensuring necessary concentration of RNA and a stable environment. Generally, we identify compartmentalization state to (C,C) with probability measure ν. Let $Q_{c}$ be a transition probability kernel from (C,C) into (E,E), encoding the transition from compartmentalization coordinates to RNA sequences. The product space (C×E,C⊗E) has law $\mu =\nu \times Q_{c}$. Upon hitting a high-fidelity replicator and achieving Darwinian evolution to acquire information, e.g., genes, the sequences are assumed to become adapted to compartmentalization coordinates (C,C) through the transition probability kernel $Q_{c}^{\prime }$ from (C×E,C⊗E) into (C,C), so that $\mu =\nu \times Q_{c}\times Q_{c}^{\prime }$ is the law on the full space (C×E×C,C⊗E⊗C). In this telling, compartmentalization precedes RNA activity, and, upon hitting high-fidelity replicators that can maintain their information, is followed by genomic adaptation.

### 2.7. Metabolism

We identify metabolism reaction-state to the measurable space (M,M) with probability measure ν. Let $Q_{m}$ be a transition kernel from (M,M) into (E,E), positing that metabolism precedes replication. For example, certain metabolic state may be precursors to the synthesis of RNA. Consider product space (M×E,M⊗E) with measure $\mu =\nu \times Q_{m}$. Now we suppose that, upon achieving Darwinian evolution in replicators, the replicators will eventually become adapted to (M,M). Hence, we interpret (M,M) as a mark-space of (M×E,M⊗E), representing genomic adaptation. Let $Q_{m}^{\prime }$ be a transition kernel from (M×E,M⊗E) into (M,M). Then, $\mu =\nu \times Q_{m}\times Q_{m}^{\prime }$ is a probability measure on (M×E×M,M⊗E⊗M), where

$$\mu \left(f\right)=\int_{M}\nu \left(dx\right)\int_{E}Q_{m}\left(x,dy\right)\int_{M}Q_{m}^{\prime }\left(\left(x,y\right),dz\right)f\left(x,y,z\right)\;\mathrm{for}\;f\in {\left(M⊗E⊗M\right)}_{\geq 0}$$

or

$$\mu \left(dx,dy,dz\right)=\nu \left(dx\right)Q_{m}\left(x,dy\right)Q_{m}^{\prime }\left(\left(x,y\right),dz\right).$$

Therefore, metabolism-first followed by replication and genomic adaptation is encoded by the structures of $Q_{m}$ and $Q_{m}^{\prime }$. We do not specify these transition kernels in this article but mention that they are richly textured.

### 2.8. Reaction Overview

The reactions of the system having decay and clay and their reaction orders are shown in Table 2. There is one zero-order reaction, three first-order reactions, and two second-order reactions. Additionally, we show reactions and orders for compartmentalization and metabolism.

## 3. Results

Consider some initial population of *I* random sequences $X_{0}$. The population over time is given by $X_{t}$ with associated random counting measure $N_{t}$ on $\left(\bar{G},2^{\bar{G}}\right)$. Recall parameters

$$\theta =(n,q,k,l,m,p,k_{⌀},k_{ss},k_{ds},k_{rep},k_{clay-o},k_{clay-p})$$

for sequence dimension *n*, high-fidelity sequence set size *q*, fitness degree *k*, similarity degree *l*, fidelity degree *m*, clay fidelity probability *p*, RNA decay rate $k_{⌀}$, double-strand dissociation rate $k_{ss}$, double-strand formation rate $k_{ds}$, RNA replication rate $k_{rep}$, and clay oligomerization and polymerization rates $k_{clay-o}$ and $k_{clay-p}$. These parameters are summarized in Table 3.

The following is the description of how the parameter values were specified and to what they biologically correspond. The sequence dimension *n* is chosen from {3,4,5}. The fitness and similarity functions are chosen by setting the value of the range of the curvature parameters *k* and *l* from one (inside the high-fidelity manifold) to some small values, such as over an exponential grid. For example, when i=0.1, the fitness of sequences that are maximally dissimilar have 10% of the fitness of the high-fidelity sequences. We range the grid from 0.1 to 0.001 for fitness and similarity. The RNA fidelity parameter m=0.25 is chosen such that the high-fidelity sequences have value one and the lowest fidelity sequences have value 0.25, equal to random chance. The clay fidelity parameter is set to an optimistically high value of 0.9 for clay studies. The double-strand dissociation and formation rates $k_{ss}$ and $k_{ds}$ are set to unity as a baseline. In comparison, the RNA replication rate is set to a large value, 10, whereby replication is the dominant reaction. The decay parameter $k_{⌀}$ is set to some uniform random value in (0, 1). The clay RNA oligomerization rate is set to unit, and ‘clay’ RNA polymerization rate is set to a uniform random value in (0, 20).

With the parameters governing the reaction rates, different values of these parameters confer different regimes for the system.

### 3.1. Stability: ODEs

We characterize the zeros of the vector field *f* from ODE system (A1) and use the eigenvalues of the Jacobian (A2) to determine their stability.

**Theorem** **2.** *The ODE system* (A1) *for R={x}, x∈E, has a single unstable fixed-point at [x]=1 and [y]=0 for y∈G\x.*

**Proof.**  Solving f=0 gives a single solution [x]=1 and [y]=0 for y∈G\x. For this solution, the eigenvalues of the Jacobian contain no zero values and positive values. Therefore, the solution is unstable. □

It follows from Theorem 2 that, for all other initial conditions, the system has no equilibria.

**Corollary** **2** (Unbounded). *For all initial conditions $X_{0}$ such that $I=|X_{0}|>1$, the system is unbounded.*

This confirms the obvious: the system, a replicating network with no death, is almost always an increasing system.

### 3.2. Simulation Reaction State

We are interested in the behavior of temporal probability measures, $\nu_{t}$ (25) on the sequence product space and $\mu_{t}=\nu_{t}Q$ (27) on the sequence space, for RNA polymerization. These reveal the instantaneous information of the system. The structure of $\mu_{t}$ reveals the state of polymerization and is a leading indicator of the population concentrations over time.

#### 3.2.1. Core Model with “Tent” Functions, Probable Hitting $P(\tau_{v}\left(\theta \right)<\infty )∼1$

**Figure 1:**
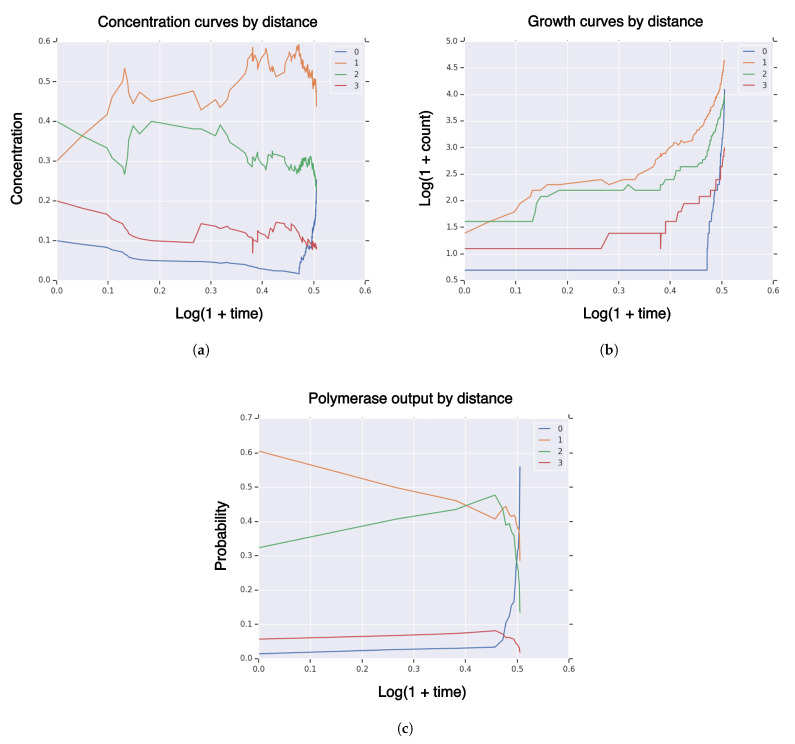
Measures of system population $X_{t}$ until hitting time $\tau_{v}$ for high-fidelity replicator volume fraction v=0.25 with sequence dimension n=3, fitness/similarity curvature l=k=−log(0.01)/n, initial population size $I=|X_{0}|=10$, singleton high-fidelity replicator R={{x}}, with “tent” fitness and similarity functions. (**a**) concentration of RNA sequences by Hamming distance to high-fidelity replicator; (**b**) population size of RNA sequences by Hamming distance to high-fidelity replicator; (**c**) polymerase RNA sequence output by Hamming distance to high-fidelity replicator.

Take sequence dimension n=3 and fitness and similarity curvature parameters k=l=−log(0.01)/n and fidelity parameter m=−log(0.25)/n. Set rates for double-strand dissociation and formation $k_{ss}=k_{ds}=1$ and polymerization rate $k_{rep}=10$ and use the “tent” function for fitness, similarity, and fidelity. Take random initial population $X_{0}$ with initial population size $I=|X_{0}|=10$ and random singleton R={{x}} (q=0). We simulate 5000 reactions, simulation censored at hitting time $\tau_{v}$ for volume fraction v=0.25. Take partition of the sequence space by Hamming distance to the high-fidelity manifold $\left(H_{i}\right)$ (28) of sequence space *E*. In Figure 1, we plot measures of a typical realization of the system $X_{t}$ on the partition $\left(H_{i}\right)$ of sequence concentration (Figure 1a), growth (Figure 1b), and polymerase sequence output $\mu_{t}$ (Figure 1c). Some quantities are plotted on log-log scale, whereas others are plotted on a linear-log scale. These results show that the concentrations are relatively stable for most time, until the high-fidelity manifold is hit. Then, the concentration of high-fidelity replicators rapidly increases to exceed 25%. Similarly, Figure 1b shows the growth curves on a log-log scale, where the high-fidelity manifold rapidly increases near the end of the simulation. Figure 1c shows the structure of the RNA sequence polymerization output temporal probability measure $\mu_{t}$. Low probability is assigned to polymerization of high-fidelity replicators for most of the reaction time, followed by a large increase near the end of the simulation, where high-fidelity replicators dominate with 56% probability. *Therefore, the RNA sequence polymerization output temporal probability measure $\mu_{t}$ is a leading indicator of the concentration curve, i.e., at simulation end-time, concentration of high-fidelity replicators is 25% and polymerization output is 56%.*

#### 3.2.2. Core Model with “Tent” Functions, Improbable Hitting $P(\tau_{v}\left(\theta \right)<\infty )∼0$

**Figure 2:**
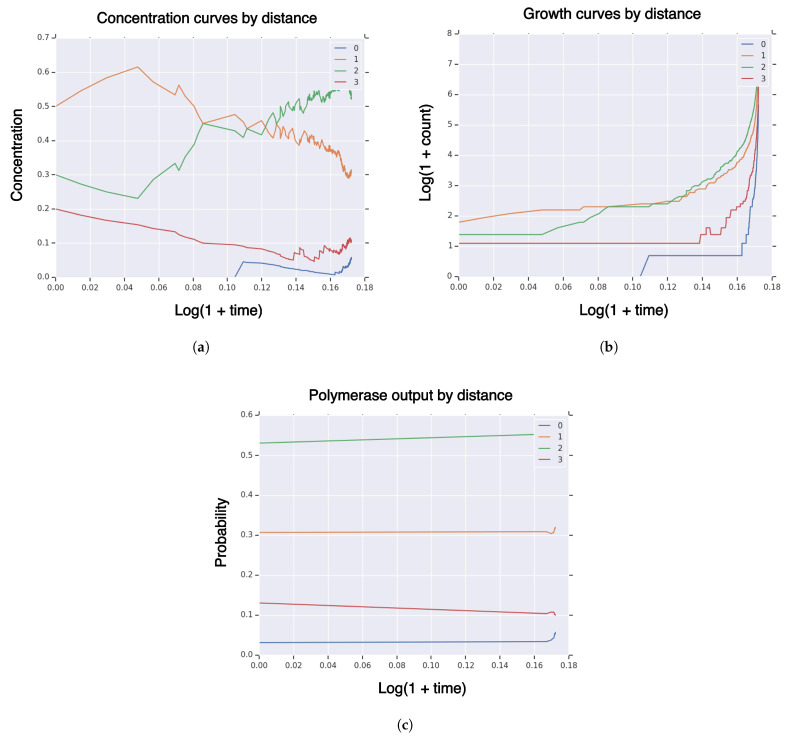
Measures of system population $X_{t}$ until hitting time $\tau_{v}$ for high-fidelity replicator volume fraction v=0.25 with sequence dimension n=3, fitness/similarity curvature l=k=−log(0.1)/n, initial population size $I=|X_{0}|=10$, singleton high-fidelity replicator R={{x}}, with “tent” fitness and similarity functions. (**a**) concentration of RNA sequences by Hamming distance to high-fidelity replicator; (**b**) population size of RNA sequences by Hamming distance to high-fidelity replicator; (**c**) polymerase RNA sequence output by Hamming distance to high-fidelity replicator.

We use the same configuration as Section 3.2.1 except for setting fitness and similarity curvature parameters to k=l=−log(0.1)/n. In Figure 2, we plot measures of a typical realization of the system $X_{t}$ on sequence partition by Hamming distance to the high-fidelity manifold $\left(H_{i}\right)$ of concentration (Figure 2a), growth (Figure 2b), and $\mu_{t}$ (Figure 2c). The behavior has completely changed: the high-fidelity group ends the simulation with around 6% concentration, only steadily increasing, and never hits. The polymerase output $\mu_{t}$ shows 6%. This indicates that the concentration of high-fidelity replicators is unlikely to increase further, as the population is generally in equilibrium with the polymerase output.

#### 3.2.3. Core Model with Linear Functions, Improbable Hitting $P(\tau_{v}\left(\theta \right)<\infty )∼0$

The same configurations for Section 3.2.1 are used, except the fitness, similarity, and fidelity functions are linear. Similar to the “tent” functions, we specify the terminus landscape curvature for fitness and similarity k=l. Then, the fitness function for RNA polymerization is given by

$$f_{k}\left(x,R\right)=1+\left(\frac{k-1}{n}\right)H\left(x,R\right)\;\mathrm{for}\;x\in E$$

and

$$s_{k}\left(x,y\right)=1+\left(\frac{k-1}{n}\right)S\left(x,y\right)\;\mathrm{for}\;x,y\in E$$

**Figure A1:**
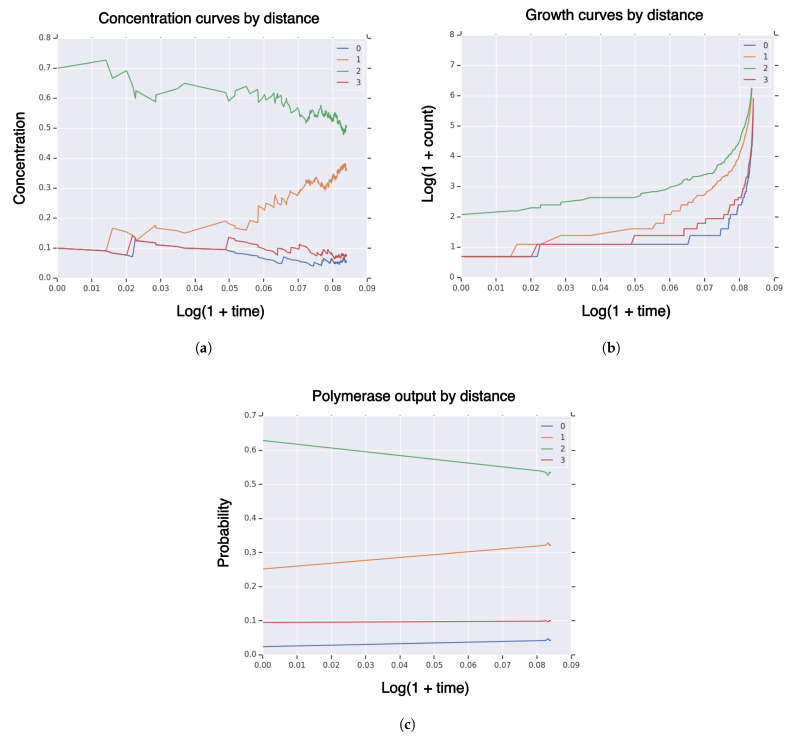
Linear landscape: Measures of system population $X_{t}$ until hitting time $\tau_{v}$ for high-fidelity replicator volume fraction v=0.25 with sequence dimension n=3, fitness/similarity curvature k=l=−log(0.01)/n, initial population size, $I=|X_{0}|=10$, singleton high-fidelity replicator R={{x}}, with linear fitness and similarity functions. (**a**) Concentration of RNA sequences by Hamming distance to high-fidelity replicator; (**b**) population size of RNA sequences by Hamming distance to high-fidelity replicator; (**c**) polymerase RNA sequence output by Hamming distance to high-fidelity replicator.

We put fitness and similarity landscape curvature k=l=0.01 for fitness and similarity and v=0.25 for hitting volume fraction of high-fidelity replicators. We simulate $X_{t}$ for 5000 reactions. We find that the probability of hitting is near zero $P(\tau_{0.25}\left(\theta \right)<\infty )∼0$. In Figure A1, we plot measures of a typical realization of $X_{t}$ on $\left(H_{i}\right)$ of concentration (Figure A1a), growth (Figure A1b), and $\mu_{t}$ (Figure A1c). The simulation ends with high-fidelity concentration of ∼5% and polymerase output of ∼4%. Therefore, the concentration of high-fidelity replicators will continue to decrease. *Linear surfaces are not sufficient to achieve hitting times $\tau_{v}\left(\theta \right)<\infty$ for high-fidelity replicator volume fraction v=0.25, in contrast to the nonlinear “tent” functions.*

#### 3.2.4. Expanded Model with “Tent” Functions, Probable Hitting $P(\tau_{v}\left(\theta \right)<\infty )∼1$

**Figure A2:**
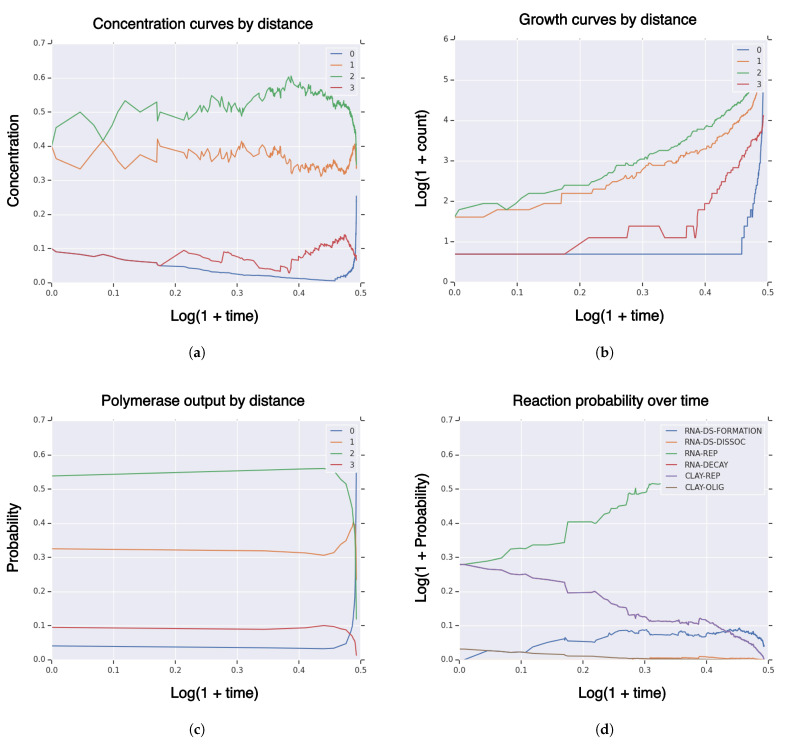
Core model with clay: Measures of system population $X_{t}$ until hitting time $\tau_{v}\left(\theta \right)$ for high-fidelity replicator volume fraction v=0.25 with sequence dimension n=3, fitness/similarity curvature k=−log(0.01)/n, initial population size $I=|X_{0}|=10$, singleton high-fidelity replicator R={{x}}, double strand separation and formation rates reaction rate $k_{ss}=k_{ds}=1$, clay replication fidelity probability p=0.9, and RNA polymerization rate $k_{rep}$ and clay polymerization rate $k_{clay-p}$ chosen such that the replicative mass of each is 10. (**a**) Concentration of RNA sequences by Hamming distance to high-fidelity replicator; (**b**) population size of RNA sequences by Hamming distance to high-fidelity replicator; (**c**) polymerase RNA sequence output by Hamming distance to high-fidelity replicator; (**d**) probability of reactions over time.

We consider a similar model to the previous subsections and expand it with clay oligomerization rate (of RNA) $k_{clay-o}$, clay polymerization rate (of RNA) $k_{clay-p}$, and clay polymerization fidelity *p*. Therefore, the full set of variables is given by $\theta =(n,k_{ss},k_{ds},k,k_{clay-o},k_{clay-p},p)$. The value of fitness/similarity landscape curvature k,l and clay RNA polymerization raet $k_{clay-p}$ are set such that the replicative mass of each is initialized to 10. This means that RNA and clay polymerization have the same reaction mass at the beginning of the simulation. We set sequence dimension n=3, fitness/similarity landscape curvature k=l=−log(0.01)/n, clay RNA polymerization fidelity p=0.9, and double-strand dissociation and formation rates $k_{ss}=k_{ds}=1$. This is a high hitting regime, i.e., the probability of hitting is close to one $P(\tau_{v}\left(\theta \right)<\infty )∼1$. In Figure A2, we plot measures of a typical realization of $X_{t}$ on $\left(H_{i}\right)$ and additionally the probability of reactions over time. High-fidelity replicators ended the simulation with 25% concentration (Figure A2a) and RNA polymerase output ∼62% (Figure A2c), indicating that the concentration of high-fidelity replicators will continue to increase. All species exhibit superexponential growth (Figure A2b). Clay polymerization decreases in contribution over time, whereas RNA polymerization increases substantially over time, and RNA double-strand reactions are small and stable (Figure A2d).

### 3.3. Hitting Times: Functional and Survival Analysis

We study various models in order of increasing complexity. We examine the hitting time surface $\tau_{v}\left(\theta \right)$ in the parameters θ∈Θ, including probability of hitting $P(\tau_{v}\left(\theta \right)<\infty )$. We begin with the core model with no decay or clay.

#### 3.3.1. Core Model, $\tau_{v}\left(\theta \right)$ for v=0.1 with θ=(n,k) and “Tent” Functions

We are interested in the structure of the hitting time $\tau_{v}\left(\theta \right)$ of (19) as a function of the parameter vector θ. We use the Weibull–Cox proportional hazard’s model of Table 1 for the hitting time $\tau_{v}$ for volume fraction v=0.1. Let θ=(n,k) with sequence dimension n∈{3,4} and fitness and similarity parameters k=l=−log(i)/n for i∈{0.1,0.05,0.01,0.005,0.001}. Set m=−log(0.25)/n for fidelity probability parameters. For each value of sequence dimension *n*, take random initial population $X_{0}$ with initial population size $I=|X_{0}|=10$ and random singleton R={{x}} for the high-fidelity manifold and fix these for the fitness/similarity landscape curvature parmeters k=l. We fix the double-strand dissociation and formation rate parameters $k_{ss}=k_{ds}=1$ and set RNA polymerization rate $k_{rep}$ such that the overall RNA polymerization rate is given by ${\overset{˘}{k}}_{rep}\left(0\right)=10$ and use the “tent” function for fitness, similarity, and fidelity. We take 10 realizations of hitting time $\tau_{v}\left(\theta \right)$ for each parameter vector θ∈Θ and allocate 5000 reactions. This gives 100 independent hitting times and up to 500,000 reactions. The times are comparable because the system is initialized to the same replication mass.

**Figure A3:**
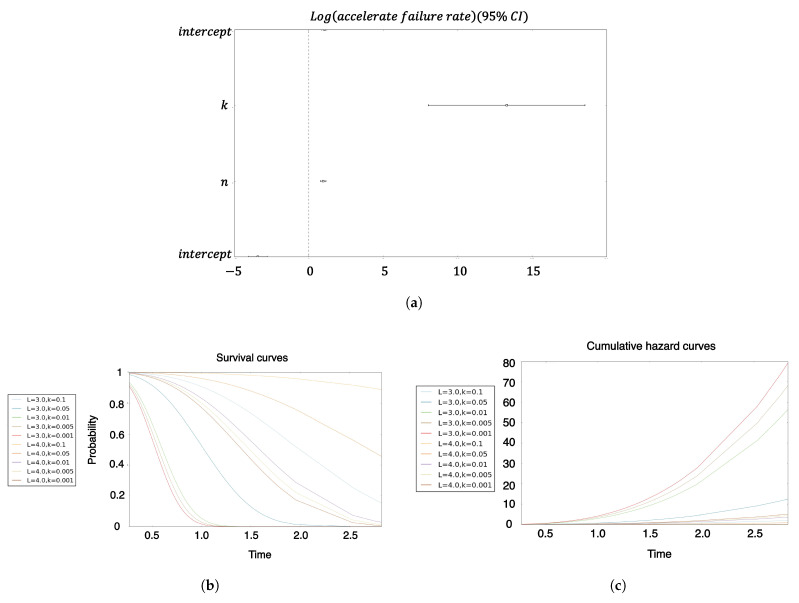
Survival analysis of hitting time $\tau_{v}$ for high-fidelity replicator volume fraction v=0.1 for core model. (**a**) Coefficients of the Cox proportional hazard survival model; (**b**) survival curves in sequence dimension n=L and landscape curvature k=l; (**c**) cumulative hazard in sequence dimension n=L and landscape curvature k=l.

For the simulations, 66 hitting times are finite. The coefficients positively contribute to hitting, where $\gamma_{n}\approx 0.97$ and $\gamma_{k}\approx 13.29$, both with *p*-values less than 0.005. Therefore, hittings are strongly positively influenced by the parameters of the fitness and similarity functions and less so by the dimension. Plots of the coefficients and survival and cumulative hazard curves are given in Figure A3. *High survival is found for k large and low survival for k small. Cumulative hazard is highest for k small.*

We estimate HDMR of the classifier (whether or not hitting time is finite) using all 100 samples. The results are shown in Figure A5 and Table 4. The HDMR explains roughly 80% of variance. $S_{k}\approx 0.69$ and $S_{n}\approx 0.06$, so *hitting probability is strongly influenced by fitness landscape curvature k and less so by sequence length n*. The component functions $f_{k}$ and $f_{n}$ for fitness landscape curvature and sequence dimension are strictly decreasing, where larger fitness landscape curvature parameter *k* results in decreasing hitting probability. These results are consistent with the survival analysis.

**Figure A4:**
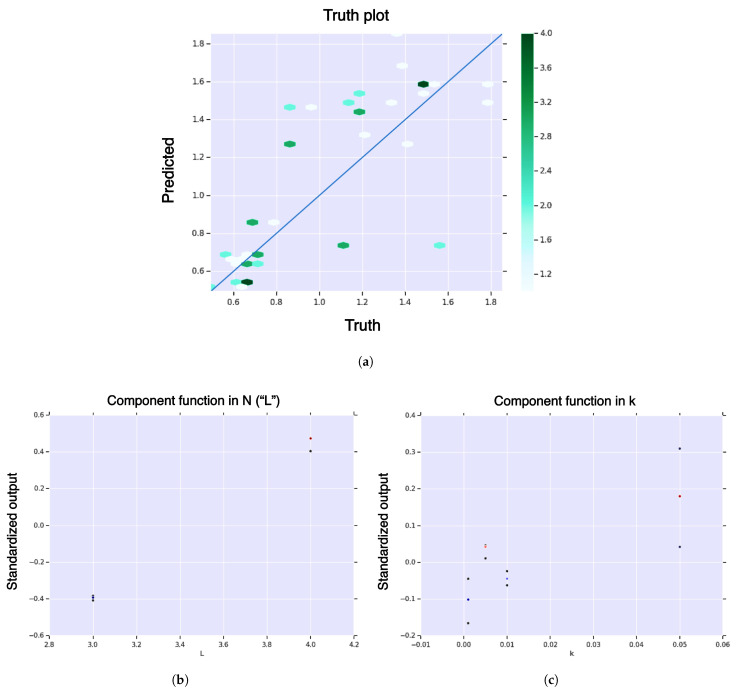
First-order HDMR analysis of hitting time $\tau_{v}\left(\theta \right)<\infty$ for core model. (**a**) Hexagonal-bin truth plot; (**b**) HDMR component function for sequence length n=L, $f_{n}\left(n\right)$, in sequence length for hitting time, of hitting time; (**c**) HDMR component function for landscape curvature, $f_{k}\left(k\right)$, in landscape curvature, of hitting time. The color function is from blue (negative) to white (zero) to red (positive). The black dots represent standard deviation of the error.

**Figure A5:**
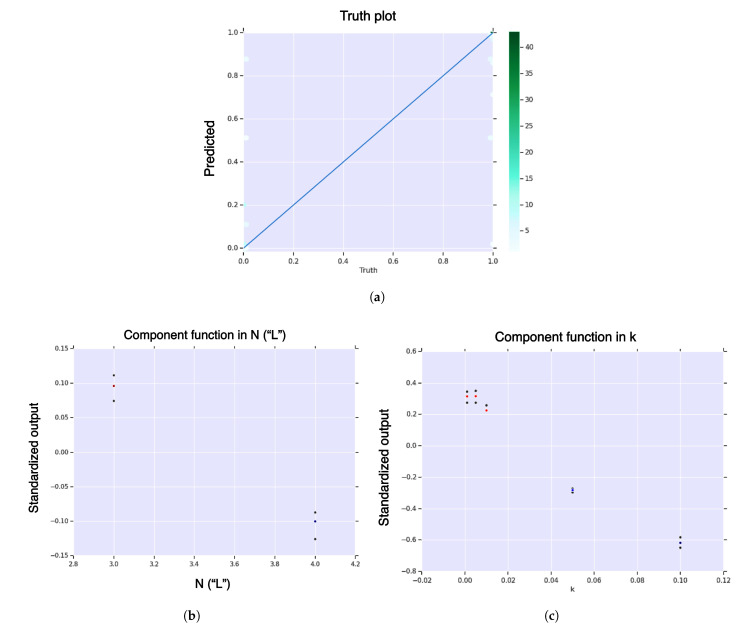
First-order HDMR analysis of hitting probability $P(\tau_{v}\left(\theta \right)<\infty )$ for core model. (**a**) Hexagonal-bin truth plot; (**b**) HDMR component function for sequence length, $f_{n}\left(n\right)$, in sequence length, of hitting probability; (**c**) HDMR component function for landscape curvature, $f_{k}\left(k\right)$, in landscape curvature, of hitting probability. The color function is from blue (negative) to white (zero) to red (positive). The black dots represent standard deviation of the error.

We estimate HDMR of the regressor (hitting time) using the 66 simulations with finite hitting time. The results are shown in Figure A4 and Table 5. The HDMR explains roughly 60% of variance. $S_{n}\approx 0.57$ and $S_{k}\approx 0.04$, so *sequence dimension dominates the hitting time*. Both HDMR component functions $f_{n}$ and $f_{k}$ for sequence dimension and fitness landscape curvature are increasing. The HDMR results reveal that conditioning on hitting reverses the roles of sequence dimension *n* and fitness landscape curvature *k*.

#### 3.3.2. Clay and Decay Model, $\tau_{v}\left(\theta \right)$ for v=0.1 with $\theta =(n,k,k_{⌀},k_{clay-p},p)$ and “Tent” Functions

We expand the model to include clay and decay. We take parameter vector

$$\begin{array}{cc} \theta & =(n,k,k_{⌀},f_{clay},p_{clay})\in \Theta \\ \Theta & =\{3,4\}\times \{-\log(i)/n:i=0.1,0.05,0.01,0.005,0.001\}\times (0,1)\times (0,1)\times (0,1) \end{array}$$

with double-strand dissociation and formation and clay oligomerization reaction rates $k_{ss}=k_{ds}=k_{clay-o}=1$. For each parameter vector θ∈Θ, (i) we choose singleton high-fidelity replicator manifold R={x} for some RNA sequence x∈E and choose random initial population of RNA molecules $X_{0}$ such that the initial population size is 10, $I=|X_{0}|=10$, and where the initial population does not intersect the high-fidelity manifold $X_{0}\cap R=⌀$, that is, the initial population does not reside on the high-fidelity manifold; (ii) we initialize the replicative mass of the system such that the initial overall RNA polymerization reaction rate is given by ${\overset{˘}{k}}_{rep}\left(0\right)=\left(1-f_{clay}\right)20$ and the initial overall clay RNA polymerization reaction rate ${\overset{˘}{k}}_{clay-p}\left(0\right)=f_{clay}20$; (iii) we sample the hitting times $\tau_{v}\left(\theta \right)$ for volume fraction of high-fidelity replicators v=0.10 a total of M=10 times, each censored by 5000 reactions, giving hitting time set T(θ) of (20). We attain input–output data set as $D=\{\left(\theta_{i},T\left(\theta_{i}\right)\right):i=1,\ldots ,240\}$. This gives a total of 2400 simulations.

**Figure A6:**
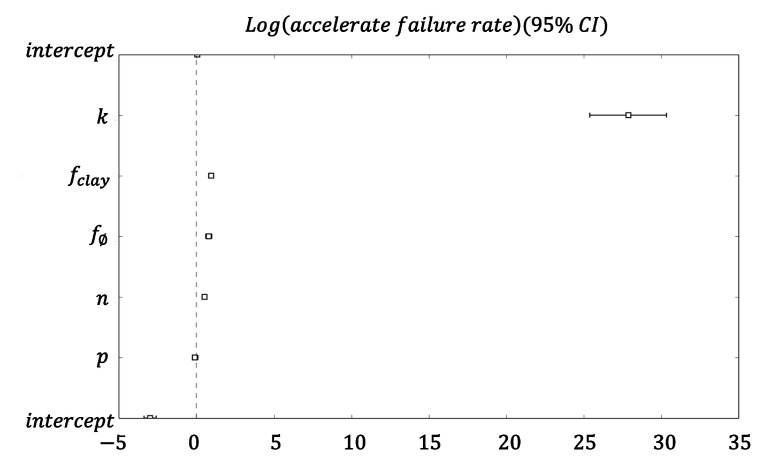
Survival analysis of hitting time $\tau_{v}$ for volume fraction v=0.1 for expanded model (clay and decay). Coefficients of the Cox proportional hazards survival model.

For the simulations, 1546 hitting times are finite. The results of fitting the Weibull–Cox model are shown below in Table 6 and Figure A6. The curvature parameter *k* again significantly dominates with a large positive value. All the remaining parameters have values less than one. Sequence dimension *n* again is a relatively small positive contributor. The clay fraction $f_{clay}$ is small and positive and replication fidelity parameter *p* is not significant.

**Figure A7:**
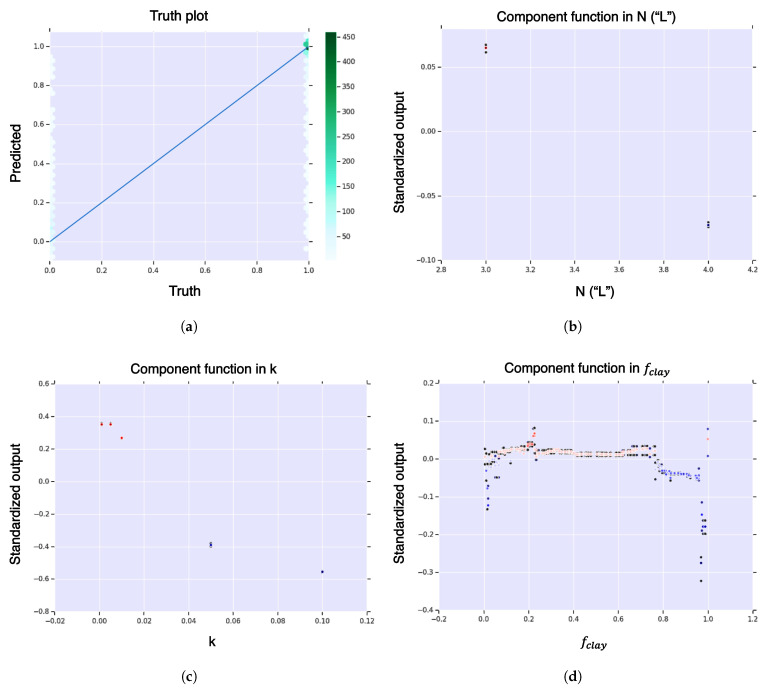
First-order HDMR analysis of hitting probability $P(\tau_{v}\left(\theta \right)<\infty )$ for expanded model (clay and decay). (**a**) Hexagonal-bin truth plot; (**b**) HDMR component function for sequence length n=L, $f_{n}\left(n\right)$, in sequence length, of hitting probability; (**c**) HDMR component function for curvature, $f_{k}\left(k\right)$, in curvature, of hitting probability; (**d**) HDMR component function for clay fitness, $f_{f_{clay}}\left(f_{clay}\right)$, in clay fitness, of hitting probability. The color function is from blue (negative) to white (zero) to red (positive). The black dots represent standard deviation of the error.

We estimate HDMR of the classifier (whether or not hitting time is finite) using all 2400 samples. Component functions and sensitivity indices are shown below in Figure A7 and Table 7. First-order HDMR captures 74% of explained variance, and second-order captures 4%. *Curvature dominates hitting probability with large sensitivity index $S_{k}\approx 67\%$*. The HDMR component function in landscape curvature $f_{k}$ is a decreasing function, where small values increase and large values decrease hitting probability. Sequence dimension *n* has sensitivity index $S_{n}\approx 2\%$, and the HDMR component function $f_{n}$ is decreasing, where high dimension decreases the probability of hitting. Clay parameter sensitivity index is small $S_{f_{clay}}\approx 2\%$, and the HDMR component function for fractional clay RNA polymerization rate, $f_{f_{clay}}$, is decreasing, where low-to-medium clay fractions increase and high-clay fractions decrease probability of hitting. The HDMR results are consistent with the Weibull–Cox model.

**Figure A8:**
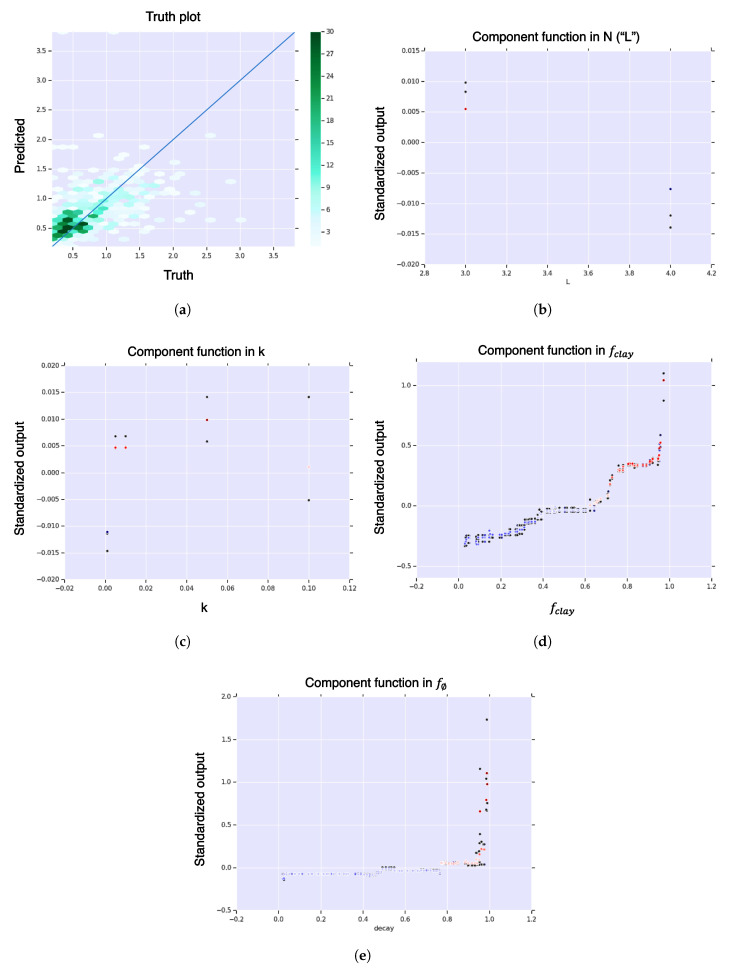
First-order HDMR analysis of hitting time $\tau_{v}\left(\theta \right)<\infty$ for expanded model (clay and decay). (**a**) Hexagonal-bin truth plot; (**b**) HDMR component function for sequence length, $f_{n}\left(n\right)$, in sequence length, of hitting time; (**c**) HDMR component function for curvature, $f_{k}\left(k\right)$, in curvature, of hitting time; (**d**) HDMR component function for clay-fraction, $f_{f_{clay}}\left(f_{clay}\right)$, in clay fraction, of hitting time; (**e**) HDMR component function for decay rate, $f_{k_{⌀}}\left(k_{⌀}\right)$, in decay rate, of hitting time. The color function is from blue (negative) to white (zero) to red (positive). The black dots represent standard deviation of the error.

We estimate HDMR of the regressor (hitting time) using the 1546 simulations with finite hitting time. Component functions and sensitivity indices are shown below in Figure A8 and Table 8. First-order HDMR captures 33% of explained variance, and second-order captures 7%. *In stark contrast to the contributions to the classifier, the parameters k and n are insignificant to hitting time. Instead, the largest sensitivity index is $S_{f_{clay}}\approx 20\%$.* The HDMR component function for fractional clay RNA polymerization rate, $f_{f_{clay}}$, is an increasing function, where small $f_{clay}$ decreases and large $f_{clay}$ increases the hitting time. This suggests that high clay-fractions representing first-order reactions increase the hitting time, as clay polymerization has less replicative mass than RNA polymerization, i.e., things go faster with RNA polymerization. The second largest sensitivity index is decay $S_{k_{⌀}}\approx 11\%$. Decay is an increasing function, with sharp increase in hitting times nearby one, i.e., things go slower with large decay resulting in increased hitting time.

### 3.4. Compartmentalization

Compartmentalization has a direct effect on the calculation of the reaction rates, specifically replication, by computing only a subset of the reactions in $X_{1}^{2}$. Put

$$X_{t}\left(A\right)=\left\{x\in X_{t}:l\left(x\right)\in A\right\}.$$

For vesicle region A∈M, we have that

$$\begin{array}{cc} {\overset{˘}{k}}_{rep}\left(t,A\right) & =\sum_{\left(x,y\right)\in X_{1}^{2}}k_{rep}\left(x,y\right)M_{t}\left(\left\{x\right\}\times A\right)\left(M_{t}\left(\left\{y\right\}\times A\right)-I_{}\left(x=y\right)\right)\;\mathrm{for}\;t\in R_{\geq 0},\;A\subset \left[-T,T\right]\\ & =\sum_{\left(x,y\right)\in X_{t}^{2}\left(A\right)}k_{rep}\left(x,y\right)M_{t}\left(\left\{x\right\}\times A\right)\left(M_{t}\left(\left\{y\right\}\times A\right)-I_{}\left(x=y\right)\right) \end{array}$$

and total replicative mass

$${\overset{˘}{k}}_{rep}\left(t\right)=\sum_{A\in M}{\overset{˘}{k}}_{rep}\left(t,A\right)\;\mathrm{for}\;t\in R_{\geq 0}$$

As M increases in size over time, the number of partitions grows, and

$$\sum_{A\in M}|X_{t}^{2}\left(A\right)|\ll |X_{1}^{2}|.$$

Therefore, the replicative mass will be reduced with M, and the system evolves less quickly. *This suggests that there is a trade-off between the degree of compartmentalization and the replicative mass of the system.*

## 4. Discussion and Conclusions

Origins of life is a fascinating problem. The wonderful complexity of extant life follows from origins. The distribution of life in the universe is tied to origins.

In this article, we have attempted to peek into the problem by concentrating on the RNA world hypothesis, studying hitting times of high-fidelity replicators. We develop fitness, similarity, and fidelity functions as landscapes for a mathematical model of replicating RNA molecules at the sequence level and observe hitting times through simulation studies. We draw attention to the distinction between the probability of hitting P(τ(θ)<∞) and the hitting time τ(θ)<∞.

In terms of mathematical set-up, we interpret the reactions as measure-kernel-functions. Each reaction is identified to and fully encoded by a probability transition kernel. The reactions take place in some domain, whereby all molecules may interact. We note that, in reality, molecules have limited diffusion, and this effectively breaks the reaction domain into independent subdomains above some length scale, i.e., molecules are more likely to react with their neighbors. Therefore, we assume our reaction volume is sufficiently small such that all molecules may participate in the reactions. We use for modeling purposes the ansatz that sequence distance is correlated to spatial proximity, where similar sequences are proximal, using a non-trivial similarity function s:E×E↦(0,1].

Theorem 2 and its Corollary 2 show that the system (without decay) is unbounded and strictly increases. This formally shows the system to be a growth process. Next, we illustrate findings about the core system with probable hitting (Section 3.2.1). In particular, we see that the temporal image measure $\mu_{t}=\nu_{t}Q$, which describes the polymerization output, is a leading indicator of high-fidelity sequence concentration. Polymerization output and high-fidelity replicators super-exponentially increase near the end of the simulation. Next, the fitness and sequence curvature parameters are set at a higher value which confers reduced fidelity (Section 3.2.2). This reveals that hitting is never achieved and that the polymerization output is in equilibrium with population composition. Hence, the probability of hitting is strongly influenced by landscape curvatures. Next, linear curvature is utilized for fitness and similarity and results in no hitting (Section 3.2.3). This reveals that nonlinear curvature is necessary to achieve hitting of high-fidelity replicators. Next, we expand the model with non-RNA (‘clay’) based polymerization and find that such activity decreases over time, in contrast to RNA polymerization, which greatly increases and dominates other reactions over time the system (Section 3.2.4).

For functional and survival analysis of the hitting times, we study the core model, whereby hitting times are strongly positively influenced by the fitness and similar functions yet are not impacted significantly by sequence dimension (Section 3.3.1). In particular, survival analysis reveals low fitness curvature confers low survival (high hitting), whereas high fitness curvature confers high survival (low hitting). HDMR analysis shows that hitting probability is strongly influenced by fitness curvature and much less so by sequence dimension, supporting the survival analysis. HDMR analysis of hitting time shows reversed roles for sequence dimension and landscape curvature, where sequence dimension dominates hitting time, with curvature playing a far less significant role. This gives the finding that hitting probability is driven by curvature, whereas hitting time is driven by sequence dimension. Next, we perform functional and survival analysis of the core model augmented with ‘clay and decay’ dynamics (Section 3.3.2). Survival analysis shows similar results to the core model, where curvature dominates survival (no hitting), with sequence dimension playing a significantly reduced role. HDMR analysis of hitting probability shows that curvature dominates hitting probability, similar to the core model, whereas sequence dimension again plays a significantly reduced role. HDMR analysis of hitting time reveals that the presence of ‘clay and decay’ significantly increase hitting time, with curvature and sequence dimension playing insignificant roles. These results are consistent in that clay polymerization has less replicative mass than RNA polymerization, where RNA polymerization is a faster reaction.

Overall, we find that *nonlinear landscapes* are *necessary* for hitting: linear landscapes are insufficient. For nonlinear landscapes, we find that the *probability of hitting* is dominated by *curvature* and that *hitting times* are dominated by *sequence dimension*. These results suggest that the landscapes in nature are nonlinear with high curvature, and that the hitting time for high-fidelity replicators is an increasing function of sequence dimension. When clay and decay are added to the model, hitting probability is again dominated by curvature, and clay and decay are relatively insignificant. This reflects that clay and decay are low order reactions. They increase hitting times.

For replication and compartmentalization, we suggest that compartmentalization, while a necessary condition, slows overall system dynamics with increasing vesiculation rate. Essentially, as compartmentalization increases, there is a corresponding reduction in absolute replicative system mass, as certain reactions among elements are no longer possible, being physically sequestered. While the timescale of a simulation is tied to the replicative system mass of the system, there is variability in replicative mass across compartments. Some compartments contain large genomic and metabolic populations. It favors the search for the high-fidelity replicator by there being a distribution on compartmental ‘fitness’ such as resource concentrations so that the high-fitness compartments drive replicative system mass. Compartmentalization is identified to the measure ν on coordinates in (C,C), for which coordinates are “marked” by sequences through the transition probability kernel $Q_{c}$, and followed by genomic adaptation via the transition probability kernel $Q_{c}^{\prime }$.

Metabolism is thought to be identified to production of precursors to RNA synthesis, leading to replication identification, followed by genomic adaptation to metabolic state. Metabolism is thus defined through the transition kernels $Q_{m}$ and $Q_{m}^{\prime }$.

The independence of the transition kernels can be scrutinized, and it is possible that general transition kernels on the full product spaces across location, genomic, and metabolic states are necessary to satisfactorily explain RNA origins, i.e., all three functions may have co-evolved. This notion is suggested in the hot springs hypothesis for origins, where compartmentalization is hypothesized to furnish necessary conditions to genomic and metabolic state [26,27,28]. In this telling, the base measurable space of interest is (F,F)=(C×E×M,C⊗E⊗M) with measure ν. Then, identification of a high-fidelity replicator is described through the transition probability kernel $Q_{cem}$ from (F,F) into (F,F) in “marking” the base measurable space with state for genomic replication; finally, genomic adaptation is conveyed through the kernel $Q_{cem}^{\prime }$ from (F×F,F⊗F) into (F,F). Hence, RNA origins of life has law $\nu \times Q_{cem}\times Q_{cem}^{\prime }$ on the product space (F×F×F,F⊗F⊗F), reflecting the steps of replicator identification and adaptation through the definitions transition kernels $Q_{cem}$ and $Q_{cem}^{\prime }$.

A putative “genesis machine” here is a mapping from the base (initial) measurable space (F,F) into the product space of identified high-fidelity replicators undergoing adaptation, i.e., (F×F×F,F⊗F⊗F). More generally the base space could additionally contain amino acid sequence space (P,P). Such a machine is fully specified through the definitions of the distribution ν on the base space and the transition kernels $Q_{cemp}$ and $Q_{cemp}^{\prime }$ (pre and post genes, respectively). Because the stages of transition occur purely through random drift, an experiment performed by such a machine would take an unacceptably long period of time to complete. Experimental demonstration can be contemplated by augmenting the base measurable space with a control space (X,X) to accelerate dynamics, using for instance closed-loop shaped radiation to address molecular degrees of freedom in their appropriate frequency domains, (open-loop) catalysts, temperature, geometry, selection, concentration through centrifugal force, etc., resulting in the new (four-dimensional) base space $\left(\tilde{F},\tilde{F}\right)=\left(C\times E\times M\times P\times X,C⊗E⊗M⊗P⊗X\right)$. Then, the transition kernels ${\tilde{Q}}_{cemp}$ and ${\tilde{Q}}_{cemp}^{\prime }$ become mappings from $\left(\tilde{F},\tilde{F}\right)$ into $\left(\tilde{F},\tilde{F}\right)$ and from $\left(\tilde{F}\times \tilde{F},\tilde{F}\times \tilde{F}\right)$ into $\left(\tilde{F},\tilde{F}\right)$, respectively. The general design of a genesis machine is the definitions of the augmented base space $\left(\tilde{F},\tilde{F}\right)$, its distribution $\tilde{\nu }$, and the augmented transition kernels ${\tilde{Q}}_{cemp}$ and ${\tilde{Q}}_{cemp}^{\prime }$, giving law $\tilde{\mu }=\tilde{\nu }\times {\tilde{Q}}_{cemp}\times {\tilde{Q}}_{cemp}^{\prime }$ on the full 15-dimensional product space $\left(\tilde{F}\times \tilde{F}\times \tilde{F},\tilde{F}⊗\tilde{F}⊗\tilde{F}\right)$, written in differential notation

$$\tilde{\mu }\left(dx,dy,dz\right)=\tilde{\nu }\left(dx\right){\tilde{Q}}_{cemp}\left(x,dy\right){\tilde{Q}}_{cemp}^{\prime }\left(\left(x,y\right),dz\right)$$

Origins could be experimentally demonstrated using a sequence of adaptive control fields in (X,X), cycling through the transitions, and a detection system for online identification of the system whose elements belong to the product measurable space. The full design and estimated operating timescale for such a machine needs further research to assess practical feasibility. We call the creation of primordial life (*primordia*) by the continuous causal efforts of a genesis machine given initial prebiotic conditions *artebiogenesis*, where *arte-* is Latin and means “from skill.” The primordia are not necessarily those that occurred in nature. Primordia and their genesis represent non-trivial system trajectories across the transition to the earliest life in sterile environments and belong to a manifold of primordial lifeforms, each having characteristic geochemical setting.

The probability measure $\mu_{t}=\nu_{t}Q$ has additional utility to integrate ‘test’ functions or queries about the system. If we let $f\in E_{\geq 0}$ be a fitness function, then the fitness value $J\left(\mu_{t}\right)=\mu_{t}\left(f\right)$ is the expected value of the fitness function with respect to the probability measure $\mu_{t}$. In OptiEvo theory, $J(\mu_{t})$ is studied as a function of the population $X_{t}$ on (E,E) [52]. OptiEvo assumes that the set of all probability measures $\left\{\mu_{t}\right\}$ is convex and that $X_{t}$ has sufficient flexibility such that $J(\mu_{t})$ may be explored around $\mu_{t}$. Then, OptiEvo predicts that $J(\mu_{t})$ has global maxima on (E,E) and that these form a connected level-set of sequences with the same fitness value. Both predictions are consistent with our model. The first prediction is consistent with zero distance in fitness and similarity functions for high-fidelity sequences. The second prediction is consistent with the high-fidelity set being a singleton or a product-space construction. A contention is whether $X_{t}$ has sufficient flexibility in exploring $J(\mu_{t})$ around $\mu_{t}$. This has direct bearing on the structure of *Q*: if $X_{t}$ is inflexible, then *Q* is constrained to certain subspaces of (E,E), i.e., not all transitions are possible.

In future work, the model could be extended to the space of sequences of lengths up to *n*, $\left(E^{*},E^{*}\right)$ or even the space of sequences of all lengths, where distance and similarity functions would utilize a more general string distance metric, e.g., Levenshtein distance. We note that the size of $\left(E^{*},E^{*}\right)$ is not much larger than (E,E). Alternative similarity functions could be explored, such as the trivial case of constant similarity, e.g., s(x,y)=1 for (x,y)∈E×E. A limitation of this article is the restriction to short sequences due to computational efficiency. The numerical size of the sequences is mathematically low-dimensional and does not correspond to actual functions of RNA molecules. Other parameter sets can be explored for example using experimentally derived values for reaction rates, so that the timescales are calibrated. Future research could see the simulation software rewritten for a high-performance computing environment, enabling much longer, e.g., length 10–1,000, sequences to be studied. Polymerase fitness can be made empirical using known RdRp sequences as members of the high-fidelity manifold. Another area of future work could be studying the aforementioned transition kernels $Q_{c}$, $Q_{c}^{\prime }$, $Q_{m}$, and $Q_{m}^{\prime }$. More general models for polymerization transition kernel based on the structure of the Poisson-binomial distribution could be employed. It would be interesting to study lipid-RNA and metabolism-RNA interactions and equip the system with the ability to append nucleotides to their sequences to form functional genes, such as storing useful information for the replication channel, perhaps a Hammerhead ribozyme to convey rolling-circle amplification. We note that transition kernels here generally lack amino acid state and are pure-RNA. An area to explore is the notion of the transition kernel into the space of high-fidelity replicators to depend on amino acid sequences and then to elaborate the system to contain a primitive translation system and examine various hitting times. Additional reactions can be introduced as operations on pairs of sequences, such as concatenation, and others for sequence splitting, and so on, with corresponding transition kernels enabling RNA networking and recombination dynamics.

## Figures and Tables

**Table 1:** Weibull reliability model.

Name	Quantity	Baseline	Quantity	Proportional Hazards
Failure density	f(t|α,β)	$\frac{\alpha }{t}{\left(\frac{t}{\beta }\right)}^{\alpha }e^{-{\left(\frac{t}{\beta }\right)}^{\alpha }}$	f(t,θ|α,β,γ)	$\frac{\alpha }{t}{\left(\frac{t}{\beta }\right)}^{\alpha }e^{\gamma \cdot \theta }e^{-{\left(\frac{t}{\beta }\right)}^{\alpha }e^{\gamma \cdot \theta }}$
Failure distribution	F(t|α,β)	$1-e^{-{\left(\frac{t}{\beta }\right)}^{\alpha }}$	F(t,θ|α,β,γ)	$1-e^{-{\left(\frac{t}{\beta }\right)}^{\alpha }e^{\gamma \cdot \theta }}$
Reliability distribution	R(t|α,β)	$e^{-{\left(\frac{t}{\beta }\right)}^{\alpha }}$	R(t,θ|α,β,γ)	$e^{-{\left(\frac{t}{\beta }\right)}^{\alpha }e^{\gamma \cdot \theta }}$
Cumulative hazard	H(t|α,β)	${\left(\frac{t}{\beta }\right)}^{\alpha }$	H(t,θ|α,β,γ)	${\left(\frac{t}{\beta }\right)}^{\alpha }e^{\gamma \cdot \theta }$
Hazard rate	h(t|α,β)	$\frac{\alpha }{t}{\left(\frac{t}{\beta }\right)}^{\alpha }$	h(t,θ|α,β,γ)	$\frac{\alpha }{t}{\left(\frac{t}{\beta }\right)}^{\alpha }e^{\gamma \cdot \theta }$

**Table 2:** Reactions and orders.

Reaction	Order
RNA double strand formation	2
RNA double strand dissociation	1
RNA polymerization	2
RNA decay	1
Clay polymerization	1
Clay oligomerization	0
Compartmentalization	1
Metabolism to replication	1
Metabolism & replication to metabolism	2

**Table 3:** Model parameters.

θ	Name	Domain	Value(s)
*n*	sequence dimension	$N_{>0}$	{3,4,5}
*k*	RNA fitness parameter	$R_{\geq 0}$	{−log(i)/n:i=0.1,0.05,0.01,0.005,0.001}
*l*	RNA similarity parameter	$R_{\geq 0}$	{−log(i)/n:i=0.1,0.05,0.01,0.005,0.001}
*m*	RNA fidelity parameter	$R_{\geq 0}$	−log(0.25)/n
*p*	clay fidelity probability	(0,1]	0.9
$k_{ss}$	double-strand dissociation rate	$R_{\geq 0}$	1
$k_{ds}$	double-strand formation rate	$R_{\geq 0}$	1
$k_{rep}$	RNA replication rate	$R_{\geq 0}$	10
$k_{⌀}$	RNA decay rate	$R_{\geq 0}$	(0, 1)
$k_{clay-o}$	clay RNA oligomerization rate	$R_{\geq 0}$	1
$k_{clay-p}$	clay RNA polymerization rate	$R_{\geq 0}$	(0, 20)

**Table 4:** HDMR sensitivity indices of hitting probability $P(\tau_{v}\left(\theta \right)<\infty )$ for the core model.

θ	$S_{\theta }$
Sequence length *n*	0.06
Curvature *k*	0.69
∑	0.75

**Table 5:** HDMR sensitivity indices of hitting time $\tau_{v}\left(\theta \right)$ for the core model.

θ	$S_{\theta }$
Sequence length *n*	0.57
Curvature *k*	0.04
∑	0.61

**Table 6:** Weibull–Cox model parameters for hitting times of clay and decay model.

θ	Name	Coefficient $\gamma_{\theta }$	*p*-Value
*n*	sequence dimension	0.54	<0.005
k=l	RNA fitness parameter	27.87	<0.005
*p*	clay fidelity probability	−0.08	0.35
$k_{⌀}$	RNA decay rate	0.80	<0.005
$f_{clay}$	fraction clay RNA polymerization rate	0.96	<0.005

**Table 7:** HDMR sensitivity indices of hitting probability $P(\tau_{v}\left(\theta \right)<\infty )$ for expanded model (clay and decay).

θ	$S_{\theta }$
Sequence length *n*	0.0213
Curvature *k*	0.6732
Decay rate $k_{⌀}$	0.0120
Clay fidelity *p*	0.0114
Fraction clay RNA polymerization rate $f_{clay}$	0.0219
∑	0.7399

**Table 8:** HDMR sensitivity indices of $\tau_{v}\left(\theta \right)<\infty$ for expanded model (clay and decay).

θ	$S_{\theta }$
Sequence dimension *n*	0.0013
Curvature *k*	0.0030
Decay $k_{⌀}$	0.1129
Clay fidelity *p*	0.0152
Fraction clay RNA polymerization rate $f_{clay}$	0.2014
∑	0.3339

**Figure A9:**
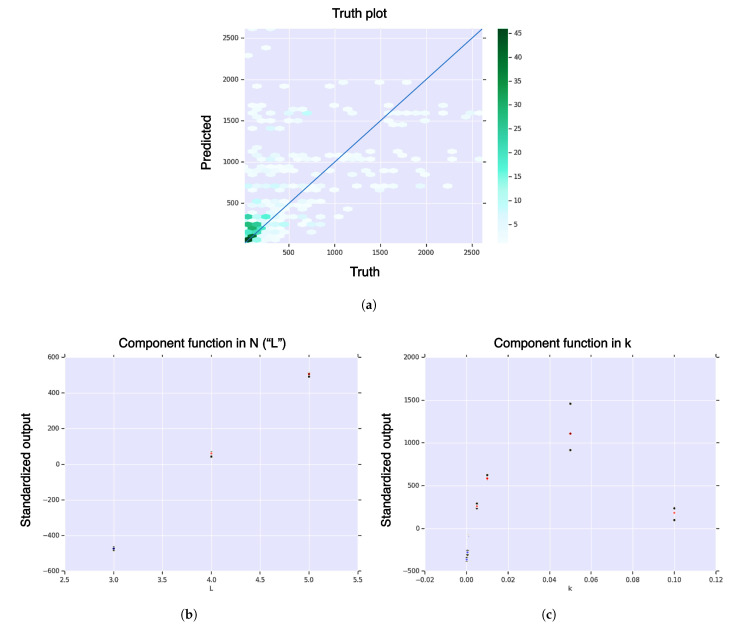
First-order HDMR analysis of hitting cardinality $\varpi_{v}\left(\theta \right)<\infty$ for core model and volume fraction v=0.1. (**a**) Hexagonal-bin truth plot; (**b**) HDMR component function for sequence dimension, $f_{n}\left(n\right)$, in sequence dimension, of hitting cardinality; (**c**) HDMR component function for curvature, $f_{k}\left(k\right)$, in curvature, of hitting cardinality. The color function is from blue (negative) to white (zero) to red (positive). The black dots represent standard deviation of the error.

## Data Availability

Please see https://github.com/calebbastian/originoflife (accessed on 31 October 2021) for the Python software and example script of usage.

## Appendix A. Discrete Probability Space

We define some concepts related to the space (E,E). The discrete probability measure ν on (E,E) is defined by

$$\nu \left(A\right)=\nu I_{A}=\sum_{x\in E}\nu \left\{x\right\}I_{A}\left(x\right)\;\mathrm{for}\;A\in E$$

where ν{x} is the probability mass at the point x∈E and

$$I_{A}\left(x\right)=\left\{\begin{array}{cc} 1 & \;\mathrm{if}\;x\in A\\ 0 & \mathrm{otherwise} \end{array}\right.$$

For the collection of non-negative E-measurable functions $E_{\geq 0}$, we have

$$\nu \left(f\right)=\sum_{x\in E}\nu \left\{x\right\}f\left(x\right)\;\mathrm{for}\;f\in E_{\geq 0}.$$

## Appendix B. Other Fitness Functions

Another fitness function can be defined using polynomials, such as lines, quadratics, etc, in terms of $k\in N_{>0}$

$$f_{k}\left(x,R\right)={\left(1-\frac{H(x,R)}{n+1}\right)}^{k}\in \left(0,1\right]\;\mathrm{for}\;x\in E$$

or

$$f_{k}\left(x,R\right)=1-{\left(\frac{H(x,R)}{n+1}\right)}^{k}\in \left(0,1\right]\;\mathrm{for}\;x\in E$$

However, another surface is using a sigmoid function. Put

$$E\left(x\right)=\frac{1}{1+\exp[-x]}\;\mathrm{for}\;x\in R$$

We have fitness for k∈(0,∞)

$$f_{k}\left(x,R\right)=\frac{1-E\left(\frac{H\left(x,R\right)-\frac{n}{2}}{k}\right)}{1-E\left(-\frac{n}{2k}\right)}\in \left(0,1\right]\;\mathrm{for}\;x\in E$$

## Appendix C. Measure-Kernel-Function

We recall a few facts about transition kernel *Q*. *Q* defines a function

$$Qf\left(x\right)=\int_{Y}Q\left(x,dy\right)f\left(y\right)\;\mathrm{for}\;x\in E$$

that is in $X_{\geq 0}$ for every function $f\in Y_{\geq 0}$.

For every probability measure $\nu_{t}$ on (E,E) and at time t≥0, the quantity $\mu_{t}=v_{t}Q$ defines a probability measure on (Y,Y) as

$$\mu_{t}\left(A\right)=\int_{X}\nu_{t}\left(dx\right)Q\left(x,A\right)\;\mathrm{for}\;A\in Y.$$

For every probability measure $\nu_{t}$ on (X,X) and function $f\in Y_{\geq 0}$, we have that

$$\mu_{t}\left(f\right)=\left(\nu_{t}Q\right)f=\int_{X}\nu_{t}\left(dx\right)\int_{Y}Q\left(x,dy\right)f\left(y\right).$$

Here, the spaces are discrete, i.e.,

$$\nu_{t}\left(A\right)\equiv \nu_{t}\left(I_{A}\right)=\sum_{x\in X}\nu_{t}\left\{x\right\}I_{A}\left(x\right)\;\mathrm{for}\;A\in X$$

where $\nu_{t}\left\{x\right\}$ is the probability mass at the point x∈X at time t≥0.

### Appendix C.1. Reactions as Measure-Kernel-Functions

We index the reaction types on $\left(Z,Z\right)=\left(N_{>0},2^{N_{>0}}\right)$. Let $\eta_{t}$ be the probability measure on (Z,Z) formed from the normalized reaction rates. Let $Q_{*}$ be the transition kernel from (Z,Z) into (X,X). Then, $\eta_{t}Q_{*}=\nu_{t}$ is the distribution on (X,X) and $\mu_{t}=\nu_{t}Q$ is the distribution on (Y,Y). Then, a reaction is the mapping (Z,Z)↦(X,X)↦(Y,Y).

### Appendix C.2. Deterministic Model

Consider the core model defined in (2)–(4) with reaction rates ${\overset{˘}{k}}_{ds}\left(t\right)$, ${\overset{˘}{k}}_{ss}\left(t\right)$, and ${\overset{˘}{k}}_{rep}\left(t\right)$. We are neglecting clay and decay for the moment. All the reactions impact (E,E). For double-strand formation, the input space is (X,X)=(E×E,E⊗E) and the output space is (Y,Y)=(F,F). For double-strand dissociation, the input and output spaces are swapped. For polymerization, (X,X)=(E×,E⊗E) and (Y,Y)=(E,E). Hence, (E,E) is positively impacted by polymerization, positively impacted by double-strand dissociation, and negatively impacted by double-strand formation. Put $\left[x\right]=N_{t}\left(\left\{\left\{x\right\}\right\}\right)$ and $\left[x,x^{c}\right]=N_{t}\left(\left\{\left\{x,x^{c}\right\}\right\}\right)$. Recall the $\nu_{t}$, *Q*, and $\mu_{t}=\nu_{t}Q$ for the reactions, e.g., $\nu_{t}^{rep},\nu_{t}^{ds},Q^{rep}$, etc.

For x∈E, we have the system of $m=\frac{3}{2}4^{n}$ deterministic nonlinear ordinary differential equations (ODEs) in *m* variables as mean-field equations

(A1) $$\begin{array}{cc} \frac{d[x]}{dt} & =f_{x}={\overset{˘}{k}}_{rep}\left(t\right)\mu_{t}^{rep}\left\{\left\{x\right\}\right\}+{\overset{˘}{k}}_{ss}\left(t\right)\mu_{t}^{ss}\left\{\left\{x\right\},\left\{x^{c}\right\}\right\}-{\overset{˘}{k}}_{ds}\left(t\right)\mu_{t}^{ds}\left\{\left\{x,x^{c}\right\}\right\}\\ & ={\overset{˘}{k}}_{rep}\left(t\right)\sum_{(y,z)\in E\times E}\nu_{t}^{rep}\left\{\left\{y\right\},\left\{z\right\}\right\}Q^{rep}\left(\left(y,z\right),\left\{\left\{x\right\}\right\}\right)+k_{ss}\left[x,x^{c}\right]-k_{ds}\left[x\right]\left[x^{c}\right]\\ & =\sum_{(y,z)\in E\times E}k_{rep}\left(y,z\right)\left[y\right]\left(\left[z\right]-I_{}\left(y=z\right)\right)Q^{rep}\left(\left(y,z\right),\left\{\left\{x\right\}\right\}\right)+k_{ss}\left[x,x^{c}\right]-k_{ds}\left[x\right]\left[x^{c}\right]\\ \frac{d[x,x^{c}]}{dt} & =f_{xx^{c}}=k_{ds}\left[x\right]\left[x^{c}\right]-k_{ss}\left[x,x^{c}\right] \end{array}$$

The fixed points of *f* are the equilibria of the system, i.e., f(x)=0 for $x\in R_{\geq 0}^{m}$. The Jacobian of the system is

(A2) $$J=\left[\begin{array}{ccc} \frac{df_{1}}{dx_{1}} & \ldots & \frac{df_{1}}{dx_{m}}\\ \vdots & \ddots & \vdots \\ \frac{df_{m}}{dx_{1}} & \ldots & \frac{df_{m}}{dx_{m}} \end{array}\right]$$

The eigenvalues of the Jacobian reveal the stability of the fixed points. If all the eigenvalues of the Jacobian evaluated at the fixed point have negative real parts, then the fixed point is stable. If none of the eigenvalues are zero and at least one of the eigenvalues has a positive real part, then the fixed point is unstable. If at least one eigenvalue is zero, then the fixed point can be either stable or unstable.

## Appendix D. Hitting Cardinality

We index the *N* reactions of $X_{t}$ with arrival times $\left\{T_{i}\right\}$ in $\left({\bar{R}}_{\geq 0},B_{{\bar{R}}_{\geq 0}}\right)$, where $\bar{R}=R_{\geq 0}\cup \left\{\infty \right\}$. Define the hitting reaction ϖ(θ)∈N in terms of hitting time $\tau \left(\theta \right)\in {\bar{R}}_{\geq 0}$ as

$$\varpi \left(\theta \right)=\sum_{i=1}^{N}I_{\left[0,\tau (\theta )\right]}\left(T_{i}\right)$$

ϖ(θ) is right-censored at *N* reactions. If τ(θ)=∞, then ϖ(θ)=N. If τ(θ)<∞, then ϖ(θ)<N.

### Reaction Cardinality

In this section, we study, instead of the hitting time $\tau_{v}\left(\theta \right)$, the hitting reaction number $\varpi_{v}\left(\theta \right)$ for v=0.1. We study the core model with parameter vector θ=(n,k). We uniformly sample sequence length n∈{3,4,5} and fitness/similarity landscape curvature k=l∈{0.0001,0.0005,0.001,0.005,0.01,0.05,0.1} and form input–output data $D=\{\left(\theta_{i},\varpi \left(\theta_{i}\right)\right):i=1,\ldots ,1200\}$. We set double-strand dissociation and formation rates $k_{ss}=k_{ds}=1$ and initialize the overall RNA polymerization rate $k_{rep}$ such that the initial replicative mass is 10. For each parameter vector θ∈Θ, we randomly choose the initial population of sequences $X_{0}$ with initial population size $I=|X_{0}|=10$ and singleton high-fidelity sequence manifold R such that the initial population of sequences does not intersect the high-fidelity replicator manifold, $X_{0}\cap R=⌀$.

D contains 781 hitting events. We denote these $D^{*}$. We form a first-order HDMR on ϖ(θ) using $D^{*}$. The HDMR truth-plot, component functions, and sensitivity indices are shown below in Figure A9. First-order HDMR captures approximately 31% of variance. Both component functions $f_{n}$ and $f_{k}$ have similar sizes, with $f_{n}$ somewhat larger than $f_{k}$. The HDMR component function for sequence dimension $f_{n}$ is essentially an increasing linear function of sequence length *n*, and component function for landscape curvature $f_{k}$ is generally an increasing function of the fitness/similarity landscape curvature. *These results suggest that sequence dimension and curvature influence the hitting reaction.* Larger sequences and flatter curvature increase the hitting reaction.

**Table A1:** HDMR sensitivity indices of $\varpi_{v}\left(\theta \right)<\infty$ for core model and v=0.1.

θ	$S_{\theta }$
Sequence dimension *n*	0.1619
Curvature *k*	0.1464
∑	0.3083

## Appendix E. Approximate Reaction Rates

One approach to reducing the computational complexity of $\overset{˘}{k}\left(t\right)$ is to approximate the sums using Monte Carlo. Define random variables $X_{1}∼\mathrm{Uniform}\left(X_{1}\right)$ and $X_{2}∼\mathrm{Uniform}\left(X_{2}\right)$. Let $\left\{X_{1i}\right\}$ and $\left\{X_{2i}\right\}$ be independencies of such random variables. Given *N* random samples of $X_{1}$, the first reaction rate becomes

$${\overset{˘}{k}}_{ds}\left(t|N\right)=\frac{|X_{1}|}{2N}\sum_{i=1}^{N}k_{ds}N_{t}\left(X_{1i}\right)N_{t}\left(X_{1i}^{c}\right)$$

whose expected value is approximated using *M* realizations,

$$E{\overset{˘}{k}}_{ds}\left(t|M,N\right)≃\frac{|X_{1}|}{2MN}\sum_{j=1}^{M}\sum_{i=1}^{N}k_{ds}N_{t}\left(X_{1ij}\right)N_{t}\left(X_{1ij}^{c}\right)$$

requiring a total of MN evaluations. In a similar manner, the second reaction rate is

$$E{\overset{˘}{k}}_{ss}\left(t|M,N\right)≃\frac{|X_{2}|}{MN}\sum_{j=1}^{M}\sum_{i=1}^{N}k_{ss}N_{t}\left(X_{2ij}\cup X_{2ij}^{c}\right)$$

and, putting $X_{1}^{*}∼\mathrm{Uniform}\left(X_{1}\right)$, we have the third reaction

(A3) $$E{\overset{˘}{k}}_{rep}\left(t|M,N\right)≃\frac{|X_{1}{|}^{2}}{MN}\sum_{j=1}^{M}\sum_{i=1}^{N}k_{rep}\left(X_{1ij},X_{1ij}^{*}\right)N_{t}\left(X_{1ij}\right)\left(N_{t}\left(X_{1ij}^{*}\right)-I_{}\left(X_{1ij}=X_{1ij}^{*}\right)\right)$$

We refer to SSA simulation with Monte Carlo approximate reaction rates as Monte Carlo Approximate SSA, or MCASSA.

## Appendix F. High Dimensional Model Representation

Suppose we have a real-valued square-integrable function $f\left(x\right)\in L^{2}\left(E,E,\nu \right)$ with $E=R^{n}$, $E=B_{R^{n}}$, $\nu =\prod_{i}\nu_{i}$, and $x=(x_{1},\ldots ,x_{n})\in E$. The $\left\{\nu_{i}\right\}$ may be diffuse (continuous) and/or discrete. Put B={1,…,n}. We would like to decompose *f* into orthogonal function subspaces $\left\{f_{u}:u\subseteq B\right\}$ (projections) in such a way that each projection on an input subspace $f_{u}$ maximizes variance and across subspaces retrieves total variance, i.e., $f=\sum_{u\subseteq B}f_{u}$ and $Varf=\sum_{u\subseteq B}Varf_{u}$. The solution to this problem in the retrieval of $\left\{f_{u}:u\subseteq B\right\}$ is known as *high dimensional model representation* (HDMR) or functional ANOVA expansion and for *f* is written as

$$f\left(x_{1},\ldots ,x_{n}\right)=f_{0}+\sum_{i}f_{i}\left(x_{i}\right)+\sum_{i<j}f_{ij}\left(x_{i},x_{j}\right)+\ldots +f_{1\ldots n}\left(x_{1},\ldots ,x_{n}\right)$$

where the $\left\{f_{u}:u\subseteq B\right\}$ are called *component functions*. For independent inputs, the component functions are mutually orthogonal and, aside from the constant component function $f_{0}=Ef$ (order zero), have zero mean $Ef_{u}=0$ for all non-empty ($2^{n}-1$) subspaces, where

$$Varf_{u}=\int_{E}f_{u}^{2}\left(x_{u}\right)\nu \left(dx\right)<\infty \;\mathrm{for}\;u\subseteq B$$

and

$$Varf=\int_{E}{\left(f\left(x\right)-f_{0}\right)}^{2}\nu \left(dx\right)=\sum_{u\subseteq B}Varf_{u}<\infty .$$

A key principle of HDMR is that the expansion for most *f* may be truncated at low order T≪n in a *T*-order HDMR,

$$f\left(x\right)≃f^{T}\left(x\right)=\sum_{u\subseteq B:|u|\leq T}f_{u}\left(x_{u}\right)\;\mathrm{for}\;T\ll n.$$

HDMR is often used in *global sensitivity analysis* to assess input–output correlations at various orders, where the variances are normalized to define *sensitivity indices*

$$S_{u}=\frac{Varf_{u}}{Varf}\;\mathrm{for}\;u\subseteq B.$$

When the inputs are correlated $\nu \neq \prod_{i}\nu_{i}$, then the component functions may still be uniquely recovered under hierarchical orthogonality, the variance decomposes

$$Varf=\sum_{u,v\subseteq B}Cov\left(f_{u},f_{v}\right),$$

where

$$Cov\left(f_{u},f_{v}\right)=\int_{E}f_{u}\left(x_{u}\right)f_{v}\left(x_{v}\right)\nu \left(dx\right)\;\mathrm{for}\;u,v\subseteq B$$

and the sensitivity indices generalize to *structural* and *correlative* sensitivity indices [56], defined respectively as

$$S_{u}^{a}=\frac{Varf_{u}}{Varf}\;\mathrm{for}\;u\subseteq B$$

and

$$S_{u}^{b}=\frac{\sum_{v\subseteq B:u\neq v}Cov\left(f_{u},f_{v}\right)}{Varf}\;\mathrm{for}\;u\subseteq B$$

with *total* sensitivity index

$$S_{u}=S_{u}^{a}+S_{u}^{b}\;\mathrm{for}\;u\subseteq B$$

where $\sum_{u\subseteq B}S_{u}=1$. We use the total sensitivity index as a measure of variable importance and the component functions as profiles of output dependence on the input subspaces.

## Appendix G. Reliability Definitions

Given a failure distribution *f* and reliability (survival) distribution *R*, we give some relations: the cumulative failure distribution is defined as

$$F\left(t|\vartheta \right)=\int_{0}^{t}f\left(s|\vartheta \right)ds$$

where

R(t|ϑ)+F(t|ϑ)=1,

the hazard rate h(t|ϑ) is defined as

$$h\left(t|\vartheta \right)=\frac{f(t|\vartheta )}{R(t|\vartheta )},$$

the cumulative hazard is defined as

$$H\left(t|\vartheta \right)=\int_{0}^{t}h\left(s|\vartheta \right)ds,$$

and we have reliability expressed in terms of the cumulative hazard

$$R\left(t|\vartheta \right)=e^{-H(t|\vartheta )}.$$

Another useful quantity is the mean residual life

$$\mu \left(t|\vartheta \right)=\frac{\int_{t}^{\infty }R\left(s|\vartheta \right)ds}{R(t|\vartheta )}.$$
